# M7G methylated core genes (METTL1 and WDR4) and associated RNA risk signatures are associated with prognosis and immune escape in HCC

**DOI:** 10.1186/s12920-023-01614-8

**Published:** 2023-08-01

**Authors:** Rui Li, Xincheng Liu, Kaiyuan Deng, Xin Wang

**Affiliations:** 1 Jiangnan University Medical Center, WuXi, China; 2 Wuxi No.2 People’s Hospital, WuXi, China; 3 The Affiliated Wuxi No.2 People’s Hospital of Clinical College of Nantong University, WuXi, China; 4 Mingguang People’s Hospital, MingGuang, China

**Keywords:** METTL1, WDR4, Prognosis, Immune escape, HCC

## Abstract

N7 methylguanosine (m7G) has a crucial role the development of hepatocellular carcinoma (HCC). This study aimed to investigate the impact of the m7G methylation core genes (METTL1 and WDR4) and associated RNA risk signatures on HCC. we found m7G methylation core genes (METTL1 and WDR4) were upregulated in four HCC cell lines, and downregulation of METTL1 and WDR4 attenuated HCC cell proliferation, migration, and invasion. Moreover, METTL1 and WDR4 are upregulated in HCC tissues, and that there is a significant positive correlation between them. METTL1 and WDR4 were identified as independent prognostic markers for HCC by employing overall survival (OS), disease-specific survival (DSS), Progression Free Interval survival (PFI), and univariate/multivariate Cox analyses. We identified 1479 coding RNAs (mRNAs) and 232 long non-coding RNAs (lncRNAs) associated with METTL1 / WDR4 by using weighted coexpression network analysis (WGCNA) and co-clustering analysis. The least absolute shrinkage and selection operator (lasso) were used to constructing mRNA and lncRNA risk signatures associated with the METTL1 / WDR4. These risk were independent poor prognostic factors in HCC. Furthermore, we found that METTL1 / WDR4 expression and mRNA / lncRNA risk scores were closely associated with TP53 mutations. Clinicopathological features correlation results showed that METTL1 / WDR4 expression and mRNA / lncRNA risk score were associated with the stage and invasion depth (T) of HCC. To predict the overall survival of HCC individuals, we constructed a nomogram with METTL1/WDR4 expression, mRNA/lncRNA risk score, and clinicopathological features. In addition, we combined single-cell sequencing datasets and immune escape-related checkpoints to construct an immune escape-related protein–protein interaction(PPI) network. In conclusion, M7G methylated core genes (METTL1 and WDR4) and associated RNA risk signatures are associated with prognosis and immune escape in HCC.

**Supplementary Information:**

The online version contains supplementary material available at 10.1186/s12920-023-01614-8.

## Introduction

Hepatocellular carcinoma(HCC) is a common neoplasm, accounting for 80% – 90% of all primary liver cancers [1]. HCC has an poor prognosis and ranks second in terms of tumor lethality [2]. While significant progress has been made in treating HCC, prognostic markers and therapeutic targets for HCC have not improved much in the clinic [3]. Although many research reports have explored the relationship between abnormal gene expression and the development of HCC, the specific mechanism of hepatocarcinogenesis remains unclear [2, 4]. Therefore, finding and screening therapeutic targets and prognostic markers for HCC is crucial.

RNA modifications can affect all RNAs' localization, splicing, and stability [5]. N7 methylguanosine (m7G) is one of the prevalent post-transcriptional modifications of RNA, which is present in various species [6]. M7G methylation is dominated by one modification of the methyl group added to the seventh N position of messenger RNA guanine (G) by the action of methyltransferases. The m7G methylation is widely distributed in tRNAs, rRNAs, and the 5 'cap region of eukaryotic mRNAs and is vital for maintaining RNA processing metabolism, stability, export, and protein translation [7]. The methyltransferase-like 1 (METTL1) and WD repeat domain 4 (WDR4) complexes are m7G methylation coregulators [8]. Recent studies have shown that the METTL1 / WDR4 complex can promote tumor progression. Jie Chen et al. showed that tRNA m7G methylation modification mediated by METTL1 / WDR4 resulted in abnormal associated protein translation, which promoted head and neck squamous cell carcinoma progression [9]. In two other studies, the METTL1 / WDR4 mediated modification of tRNA m7G methylation promotes lung and liver cancer progression [10, 11]. However, METTL1 or WDR4 interacting mRNAs and lncRNAs have rarely been studied in liver cancer. Therefore, insight into how the METTL1 / WDR4 mediated m7G methylation modification interacts with lncRNAs and mRNAs in HCC progression could help identify practical markers and therapeutic targets.

In this study, we demonstrated through in vitro experiments that METTL1 and WDR4 can promote HCC progression as oncogenes. We then identified METTL1/WDR4-associated RNAs (mRNAs and lncRNAs) using multiple algorithms and constructed an optimized mRNA/lncRNA risk signature. The prognostic and clinical significance of the METTL1 / WDR4 and mRNA / lncRNA risk signatures in HCC were comprehensively evaluated, and a Nomogram prediction model was constructed to predict overall survival in HCC patients. In addition, We constructed a METTL1 / WDR4 immune escape-associated protein interaction network (PPI) based on single-cell sequencing data and immune escape checkpoints. This study provides a theoretical basis for prognostic and therapeutic targets for patients with HCC.

## Materials and methods

### Cell culture and cell transient transfection

A total of five cell lines were employed in this study, including one normal liver cell line (WRL68) and four liver cancer cell lines (Huh-7, MHCC97-H, SMMC-7721, and SNU449). The cells were cultured in DMEM complete medium (PONOSAY, China) at 37 °C and 5.0% CO2 incubator environment. The transfection reagent used in this study was EndoFectinTM-MAX (GeneCoeiaTM, China). When the density of hepatoma cells (SNU449) reached about 70% during cell transfection, transfection experiments were performed according to the reagent manufacturer's instructions. Si-METTL1 and si-WDR4 were constructed by china GeneCoeiaTM. Si-METTL1: GATGACCCAAAGGATAAGAAA. Si-WDR4:CAGAAAAGAAGTCACAAGAAAAT.

### Total RNA extraction and quantitative real-time polymerase chain reaction (qRT-PCR)

Total RNA was extracted in this study using the Trizol Kit (Thermo Fisher, China). Reverse transcription and quantitative fluorescent PCR were performed using HiScript III RT SuperMix for qPCR and SYBR Green PCR Master Mix Kit (Vazyme, China). The reagent manufacturer's instructions performed the operation process. GAPDH served as the internal reference gene for this experiment. The relative expression of genes was calculated using a 2- ΔΔ Cq method.Human METTL1 forward primer: 5’- GGCAACGTGCTCACTCCAA-3’. Human METTL1 reverse primer:5’-CACAGCCTATGTCTGCAAACT-3’. Human WDR4 forward primer:5’- ACAGCCCTGACTTTCATAGCC -3’. Human WDR4 reverse primer:5’- TCACAGCCACATCTAACAGCATA -3’.Human GAPDH forward primer: 5’-ATTGAAAATTCAGGATGGGCTTTT-3’. Human GAPDH reverse primer:5’- GTTTCTGGGCTTCTCTTTGGACTC-3’.

## CCK8

In this experiment, four 96 well plates were prepared and examined at 24 h, 48 h, 72 h, and 96 h. 200 UL per well of experimental wells containing 1 × 103 cells and cell viability was assessed using the CCK8 kit. Absorbance was measured using a microplate reader with 450 nm wavelength. CCK8 kit was purchased from Beyotime.

### Transwell assay

This study used Transwell chambers to evaluate liver cancer cells' (SUN449) migration and invasion abilities. First, cells were treated by starvation using an incomplete medium (without serum) the night before. Then 100 UL containing 2 × 104 cells were seeded in the upper Transwell chamber, 550 UL of medium containing 10% serum was added in the lower chamber, and finally incubated in an incubator containing 5% CO2 at 37°C for 24 h. At the end of the culture, fixed staining was performed, and six randomly selected fields were counted for the number of cells that migrated out or invaded. Invasion experiments were performed in the presence of Matrigel (BD Biosciences). The Transwell chamber model used for this experiment was 3422 and was purchased from Costar, USA.

The University of ALabama at Birmingham CANcer data analysis Portal (UALCAN).

UALCAN (https://ualcan.path.uab.edu/index.html) was used to evaluate the expression of METTL1 and WDR4 in protein levels, respectively.

### Expression data acquisition and analysis of HCC and normal liver samples

HCC expression data were obtained from The Cancer Genome Atlas (TCGA) database (http://tcga.xenahubs.net) (tumor:*n* = 374, normal:*n* = 50), Genotype Tissue Expression (GTEx) (https://www.gtexportal.org/home/index.html) (normal:*n* = 110) and International Cancer Genome Consortium (ICGC) (https://dcc.icgc.org/) database (tumor:*n* = 240, normal:*n* = 441). This study combined TCGA and GTEx data into a new TCGA_GTEX dataset (tumor:*n* = 374, normal:*n* = 160). Datasets containing HCC samples were obtained in the Gene Expression Omnibus (GEO) (https://www.ncbi.nlm.nih.gov/geo/) database using the following search terms: HCC or hepatocellular carcinoma, using (Entry type) and Homo sapiens (Organism) filters to specify search results. The required datasets were included according to the following exclusion criteria: (1) datasets containing only cell line samples; (2) less than 10 cases each in the normal and tumor groups; (3) datasets containing only HCC tissue and not normal tissue; (4) datasets without METTL1 or WDR4 expression. The expression levels of the continuous variables METTL1 and WDR4 in HCC were assessed using standardized mean difference (SMD). SMD < 0.2, mildly expressed; SMD between 0.2 and 0.8, moderately expressed; and SMD > 0.8, highly expressed [12]. METTL1 or WDR4 expression data from GEO, ICGC, and TCGA_GTEx were merged using a random effects model (P ≤ 0.1 and I2 > 50%). The combined results were presented as forest plots, and Begg's test was used to assess publication bias. Stata 14 software was used for the above analysis. In addition, differences between the two groups were analyzed and visualized using the R software "limma" and "ggplot2" packages. Characteristic Curve (ROC) curves of ICGC and TCGA_GTEx were analyzed and visualized using the R software "pROC" and "ggplot2" packages. In addition, the larger the Area Under the Curve (AUC), the better the ability to distinguish tumor from non-tumor.

### Clinical data acquisition and prognostic analysis

In the TCGA-HCC cohort, retention of HCC samples containing survival time (*n* = 370), survival status (*n* = 370), age (*n* = 370), sex (*n* = 370), Grade (*n* = 365), tumor stage (*n* = 346), Invasion depth (T) (*n* = 367), Lymph node metastasis (N) (*n* = 256), and Distant metastasis (M) (*n* = 270) of HCC samples. Prognostic analysis of METTL1/WDR4 in HCC patients included Overall Survival (OS), Disease-Specific Survival (DSS), Progress Free Interval (PFI), and univariate/multifactorial Cox regression analysis.In the ICGC cohort, HCC samples containing survival time (*n* = 231), survival status (*n* = 231), age (*n* = 261), gender (*n* = 261), and tumor stage (*n* = 261) were retained. Prognostic analysis of HCC patients included OS and univariate/multivariate Cox regression analysis. The R software "survival, "survminer, "regplot, "RMS "packages were used for differential analysis and visualization.

### Weighted gene co-expression network analysis (WGCNA) to obtain mRNA and lnRNA associated with METTL1/WDR4

WGCNA [13] was used to obtain METTL1 / WDR4-related mRNAs / lncRNAs. In this study, a co-expression network of METTL1 / WDR4 was constructed by Pearson correlation coefficient based on TCGA-HCC cohort (tumor: *n* = 370) using R software "WGCNA "package. Finally, the genes with significant modules were selected as METTL1 / WDR4 associated mRNA / lncRNA(*R* > 0.4, *P* < 0.05).

### Acquisition of differential mRNA/lnRNA based on METTL1/WDR4 subpopulation

Unsupervised consensus clustering is a k-means machine learning algorithm [14]. In this study, Unsupervised consensus clustering was used to analyze the TCGA cohort based on METTL1 and WDR4 expression. The R software package "pConsensusClusterPlus" was used for clustering. Overall survival curves were used to assess the prognosis of HCC patients in different clusters. The R software "limma," "ggpubr," and "ComplexHeatmap" packages were used to analyze the correlation of different clusters with clinicopathological characteristics. The R software "limma" and "ggplot2" packages were used to screen the differential mRNA and lncRNA between different clusters. visualization was done by volcano plot, and the screening conditions were set: |logFC|> 0.585, *P* < 0.05.

### Acquisition of mRNA and lnRNA closely related to HCC

In this study, the mRNA/lncRNA of the WGCNA results (significantly different modules) intersected with the upregulated genes (mRNA and lncRNA) in the METTL1/WDR4 subgroup. The common mRNA/lncRNA was then screened by univariate COX regression analysis. In this study, mRNAs/lncRNA related to HCC prognosis were selected and defined as mRNAs/lncRNA closely related to HCC progression. Finally, the "clusterProfiler" and "org.Hs.eg.db" packages were used for KEGG and GO enrichment analysis of mRNAs closely related to HCC progression.

### Construction of METTL1/WDR4-associated mRNA/lncRNA risk signature

Based on the mRNA/lncRNA closely related to HCC, this study constructed METTL1/WDR4-related mRNA and lncRNA risk signature. LASSO Cox regression analysis was performed using the R software packages "glmnet" and "survival" to construct the risk signature. The normalized expression levels of each gene and the corresponding regression coefficients were used to calculate patients' risk scores as follows: = ∑Coefi * Express. The R software "survival," "survminer," and "survminer" packages were used to plot the overall survival curves and time-dependent ROC curves for mRNA and lncRNA risk signatures. Principal Component Analysis (PCA) scatter plots were plotted using the R software packages "Rtsne" and "ggplot2" to distinguish between patients at risk for the risk profile. Multi-factor cox regression analysis was used to assess the prognostic value of mRNA and lncRNA risk scores for HCC patients. The above data were obtained from the TCGA cohort (tumor: *n* = 370). In addition, the TCGA_GTEX dataset was used to analyze the differential expression of mRNA and lncRNA in the risk signature.

### Comprehensive analysis of METTL1/WDR4 and mRNA/lnRNA risk signature

A predictive Nomogram was constructed and validated in this study. Nomogram was based on multivariate Cox regression analysis and was used to predict the overall survival of HCC patients at 1, 2, and 3 years. The calibration curve was used to predict the overall survival of HCC patients. TCGA HCC samples (*n* = 343) containing both ages, gender, grade, and stage were used for the above data. Subsequently, this study divided the median values of METTL1 / WDR4 expression and mRNA / lncRNA risk scores into high and low two groups. The Sangerbox 3.0 tool was used (https://doi.org/10.1002/imt2.36). Mutations in significant HCC genes (TP53、CTNNB1、ALB、AXIN2、KEAP1、BAP1、NFE2L2、LZTR1、RB1、PIK3CA、KRAS、IL6ST、CDKN2A、ARID2、ARID1A、ACVR2A、NRAS、HISR1H1C、PTEN、ERRFI1) were compared between high and low METTL1 / WDR4 expression groups and high and low mRNA / lncRNA risk groups [15], and waterfall plots present the results. Data for the Sangerbox 3.0 tool were obtained from the TCGA database. Differential analysis and visualization of the associations between METTL1 / WDR4 expression and mRNA / lncRNA risk scores and clinicopathological features were performed using the R software 'limma' and 'pheatmap' packages. In addition, the associations of METTL1 / WDR4 and mRNA / lncRNA risk signature with core genes of epithelial-mesenchymal transition (EMT) (CDH1, CDH2, VIM, SNAI1, SNAI2, TWIST1, MMP2, MMP3, MMP9, ZEB1) were assessed using R software "reshape2 "and "RColorBrewer "packages.

### Single cell sequencing data acquisition and analysis

The single-cell sequencing dataset GSE146115 [16] was obtained from the GEO database (HCC: *n* = 4). In this study, the R software "Seurat, "singler, "celldex, "and "monocle "packages were used to process the single-cell sequencing data. Principal component analysis (PCA) and t-SNE were employed for dimensionality reduction and clustering subgrouping. Annotation was performed according to the signature genes of each cell cluster. Regarding cell communication, the R software "sqjin / cellchat "package was used to analyze the crosstalk links among liver parenchymal cells, T cells, NK cells, and macrophages and to predict the receptors and ligands between them. This study further employed R software "reshape2 "and "rcolorbrewer "package to analyze the correlation of METTL1 / WDR4 and mRNA risk signature with the immune escape-related checkpoint. Subsequently, this study was based on GeneMANIA database construction (http://genemania.org/) to construct immune escape-associated protein–protein interaction(PPI) network. Nodes of this PPI include METTL1 / WDR4, mRNA risk signature genes, four cellular receptors and ligands (liver parenchymal cells, T cells, NK cells, and macrophages), immune escape-related proteins, and potential proteins with interactions with them.

## Statistical analysis

Graphpad 6.02, Stata 12.0 software, and R version 4.2.0 software were used for statistical analysis. In vitro experiments were all performed in triplicate, and statistical analysis was performed using an independent samples t-test. In other analyses, differences between the two groups were analyzed using the Wilcoxon rank sum test or paired t-test. Correlation analysis was based on the Pearson correlation test. The chi-square and Kruskal tests assessed associations between METTL1 / WDR4 expression or risk score and clinicopathological characteristics. In vitro experiments for this study were performed in three independent replicates. *P* < 0.05 was considered statistically significant. (*P < 0.05, **P < 0.01, ***P < 0.001, ****P < 0.0001).

## Results

### Downregulation of METTL1 and WDR4 could inhibit hepatocyte proliferation, migration and invasion

**Fig. 1:**
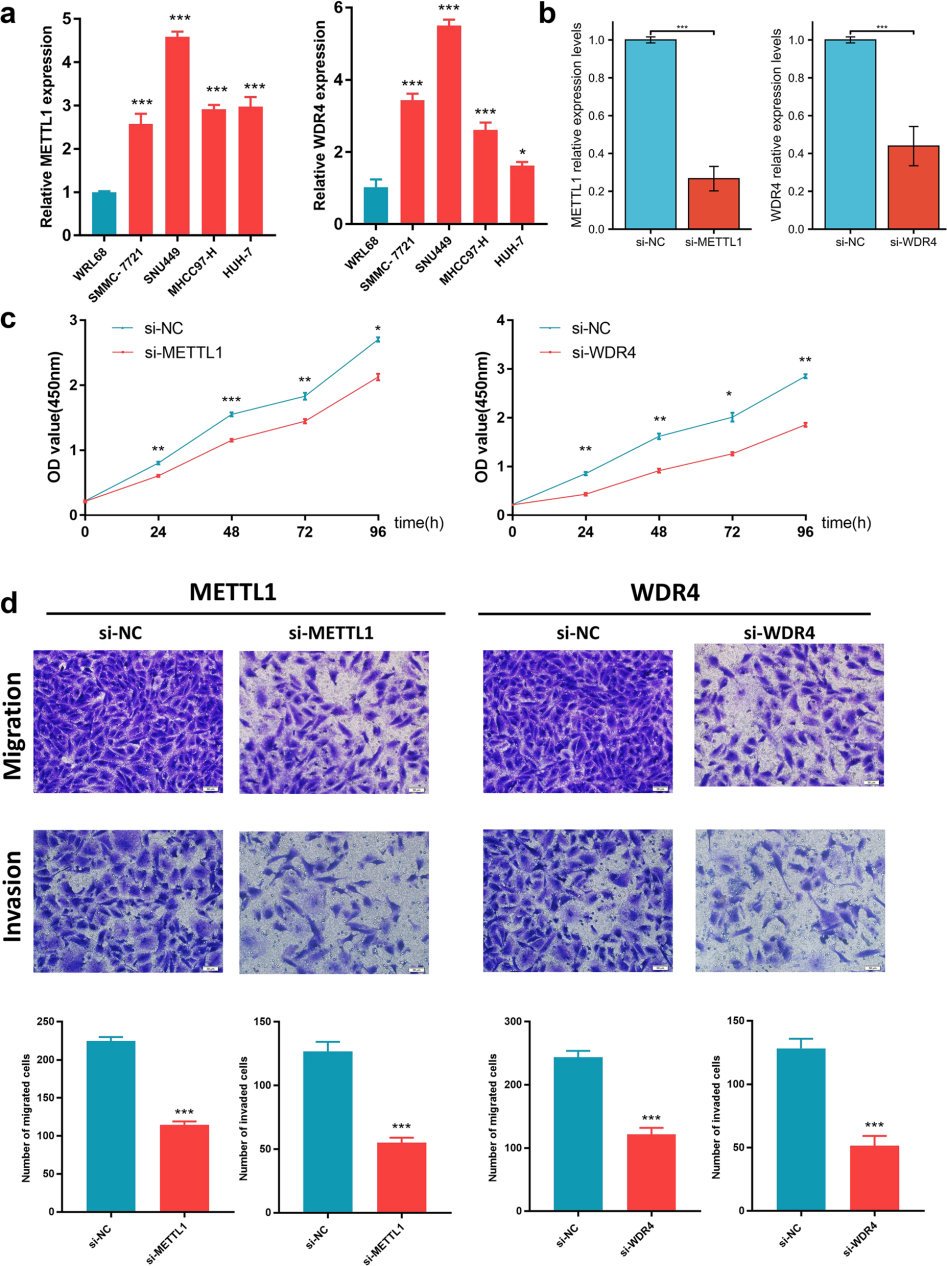
Downregulation of METTL1 and WDR4 can inhibit hepatocyte proliferation, migration and invasion. **a** The relative expression levels of METTL1 / WDR4 in a normal cell line (WRL68) and four hepatoma cell lines (Huh-7, MHCC97-H, SMMC-7721, and SNU449) were determined by RT -qPCR. **b** Transfection efficiency was determined by fluorescent quantitative PCR. **c** CCK8 experiments. **d** Transwell experiments

In order to evaluate the effects of METTL1 /WDR4 on HCC cells, the present study was evaluated by in vitro experiments. We first employed the UALCAN database to evaluate the expression of METTL1 and WDR4 in protein levels, respectively. We found that the expression of METTL1 and WDR4 protein levels were significantly elevated in HCC samples relative to normal liver tissue samples (Additional file 1: Figure S1). The qRT-PCR results showed that METTL1 and WDR4 were significantly upregulated in HCC cells (Fig. 1a), with the most significant upregulation in SNU449. Therefore, the present study transfected siRNAs (si-METTL1 and si-WDR4) into the snu449 cell line. Transfection efficiency was shown in Fig. 1b. CCK8 and Transwell experiments were subsequently performed. CCK8 results indicated that the downregulation of METTL1 and WDR4 suppresses snu449 cell proliferation (Fig. 1c). Transwell assay results indicated that downregulation of METTL1 and WDR4 also decreased SNU449 cell migration and invasion (Fig. 1d). Thus, METTL1 and WDR4 are able to promote HCC progression as oncogenes.

### METTL1 and WDR4 expression are upregulated in HCC tissues and positively correlated

**Fig. 2:**
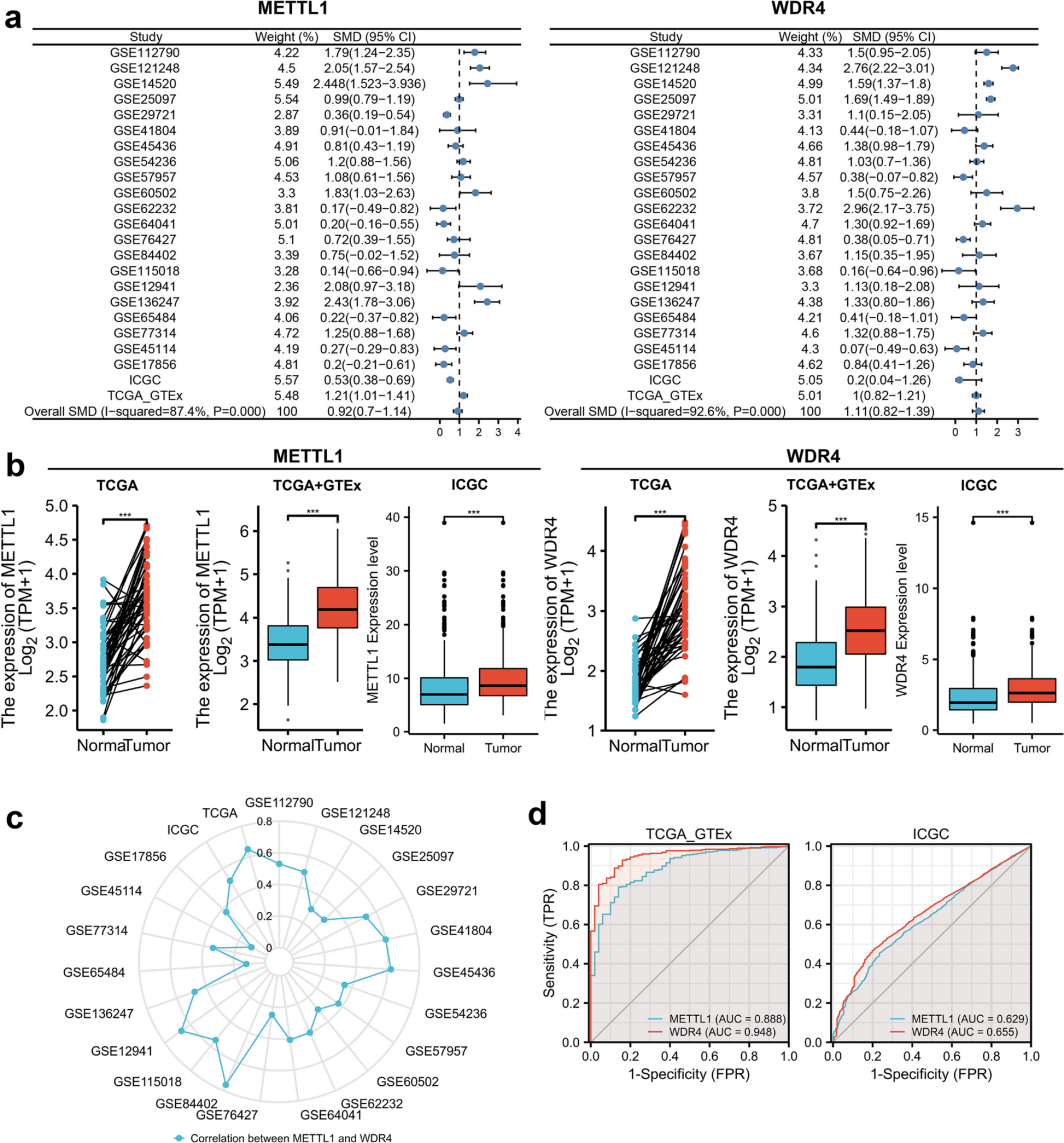
METTL1 and WDR4 expression are upregulated in HCC tissues and positively correlated. **a** Forest plot of combined SMD using random effects model. **b** Differential expression of METTL1 and WDR4. **c** Correlation analysis between METTL1 and WDR4. **d** ROC curve

Based on the exclusion criteria, 21 datasets were included in this study from the GEO database (GSE112790, GSE121248, GSE14520, GSE25097, GSE29721, GSE41804, GSE45436, GSE54236, GSE57957, GSE60502, GSE62232 GSE64041, GSE76427, GSE84402, GSE115018 GSE12941, GSE136247, GSE65484, GSE77314, GSE45114, GSE17856). Detailed information was shown in table S1 (Additional file 2). In this study, using a random effects model to combine SMD. We found that METTL1 and WDR4 were highly upregulated in HCC tissues (METTL1:SMD = 0.92, WDR4:SMD = 1.11) (Fig. 2a). In addition, Begg’s test indicated no publication bias in SMD analysis (*P* > 0.05). Subsequently, differential analysis of TCGA_GTEx and ICGC cohorts in this study showed that METTL1 and WDR4 were significantly upregulated in HCC tissues (Fig. 2b). With TCGA_GTEx, ICGC, and 21 GEO datasets, we found that METTL1 significantly correlated with WDR4 in each dataset (Fig. 2c). In addition, METTL1/WDR4 had a better ability to distinguish tumor from non-tumor (AUC > 0.6) based on ROC curves (Fig. 2d).

### Prognostic value of METTL1/WDR4 for HCC

**Fig. 3:**
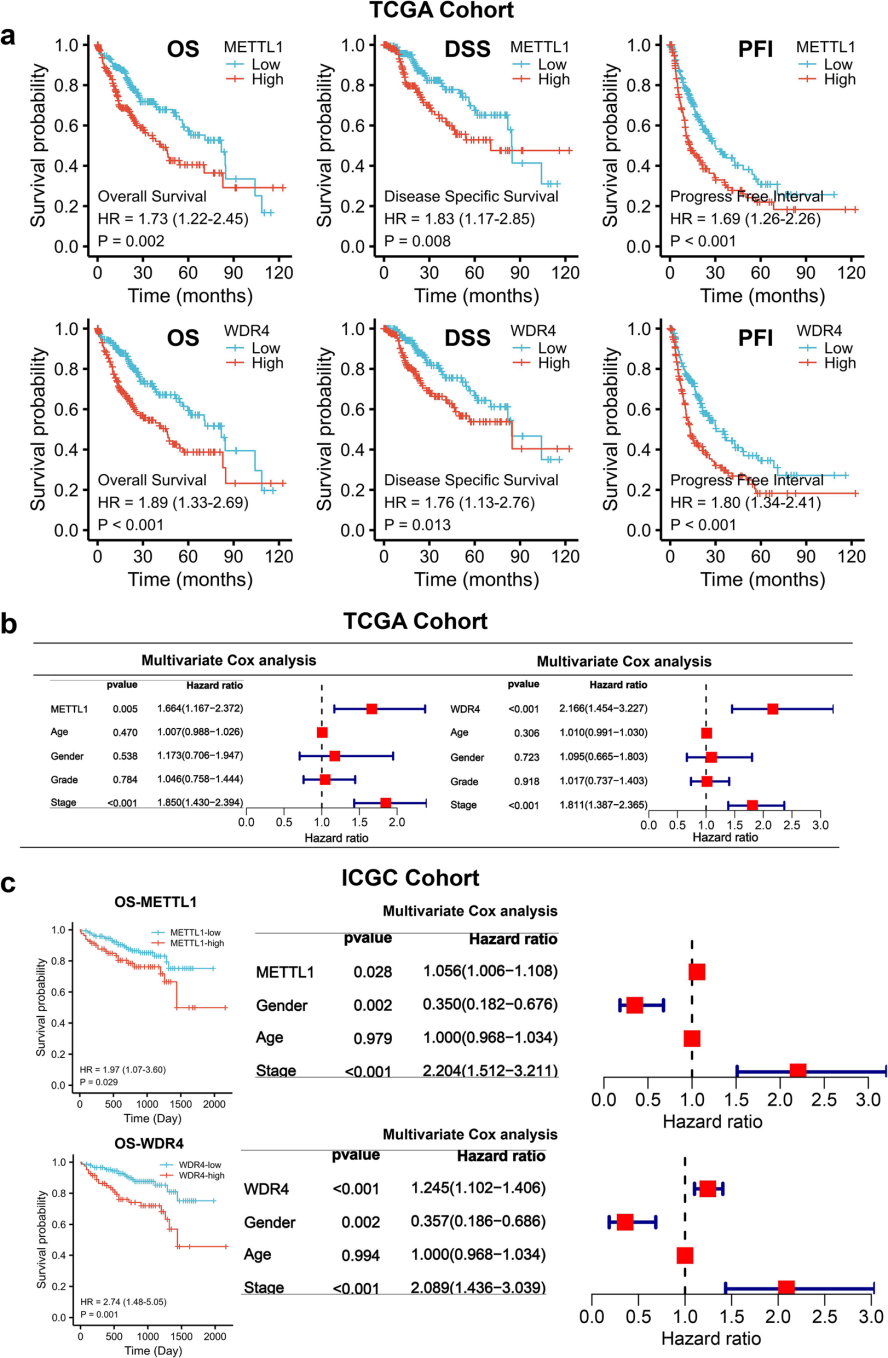
Prognostic value of METTL1/WDR4 for HCC. **a** Overall survival (OS), disease-specific survival (DSS), and progression-free interval (PFI) curves for METTL1 and WDR4. **b** Multifactorial regression analysis. **c** OS curves and multivariate regression analysis

This study evaluates the prognostic value of METTL1 and WDR4 for HCC based on TCGA and ICGC survival data. The survival results indicated that the survival of patients with high METTL1/WDR4 expression in the Overall Survival(OS), Disease Specific Survival(DSS), Progression Free Interval(PFI) curves was significantly shorter than those with low METTL1/WDR4 expression (*P* < 0.05) (Fig. 3a). In this study, METTL1 and WDR4 were combined to evaluate the prognostic effect on HCC, and we found that the high expression group of both METTL1 and WDR4 remained poor prognostic factors for HCC (Additional file 1: Figure S2). METTL1 or WDR4, age, gender, grade, and stage were included in the multivariate regression analysis in this study. Multivariate regression analysis results indicated that METTL1, WDR4, and stage were independent prognostic factors for HCC patients (Fig. 3b).The same was true for the outcome in the ICGC cohort (Fig. 3c). Since METTL1 expression, WDR4 expression, and stage are all independent prognostic factors for HCC patients. Subsequently, this study compared the expression of METTL1 or WDR4 in stages I-II, stage II-III, and stage III-IV to their effects on the survival status of HCC patients. The METTL1 survival results showed that except for the DSS survival curve in stage I—II and the PFI survival curve in stage II—III, the rest of the results showed that the survival of the METTL1 high expression group was significantly shorter than that of the METTL1 low expression group (Additional file 1: Figure S3a). The WDR4 survival results showed that, except for the DSS and PFI survival curves in stage III-IV, the rest of the results showed that the WDR4 high expression group had a significantly worse survival than the WDR4 low expression group did (Additional file 1: Figure S3b).

### Acquisition of mRNAs and lncRNA closely related to HCC

**Fig. 4:**
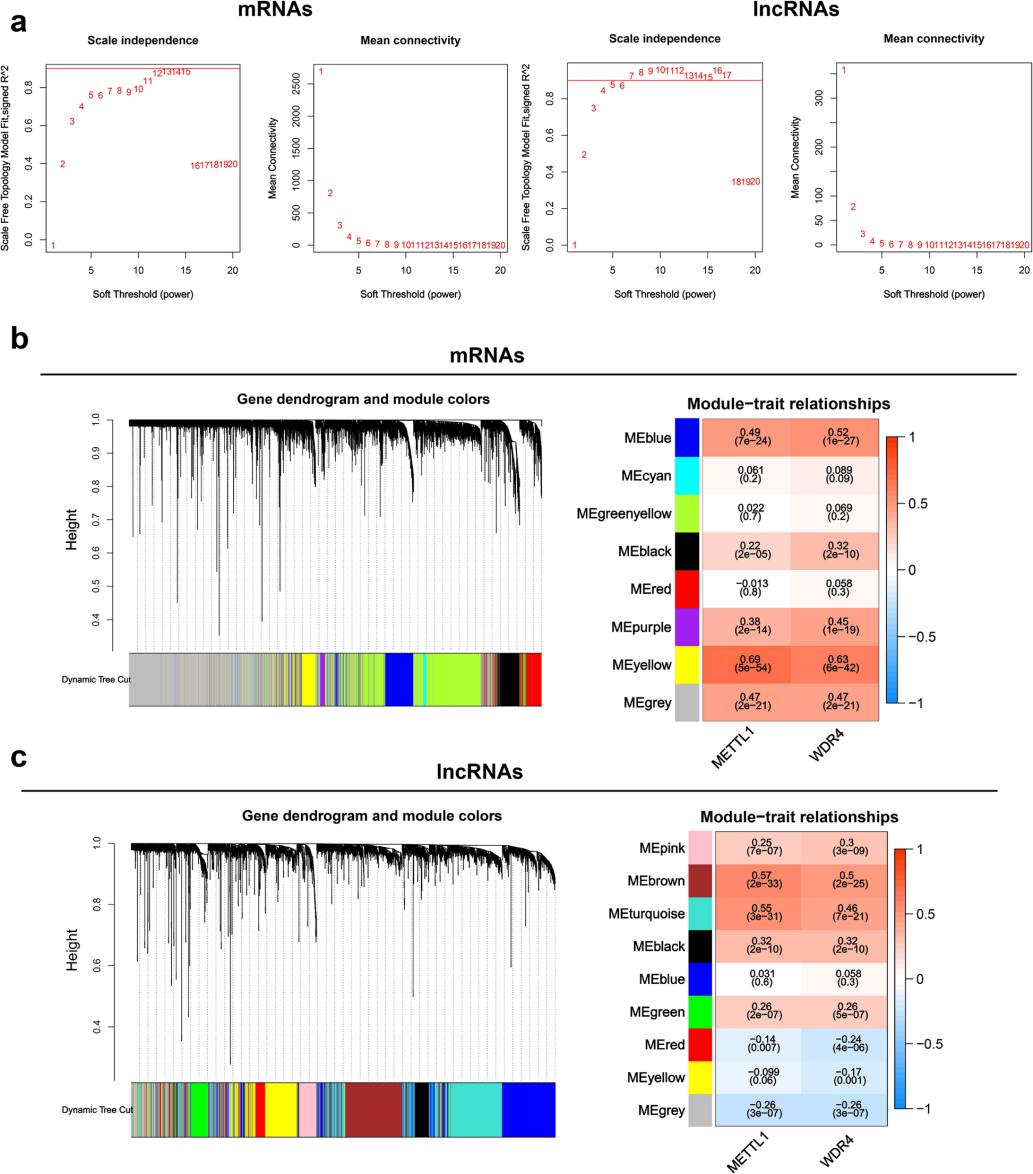
Acquisition of mRNAs / lnrnas positively correlated with METTL1 / WDR4. **a** Soft thresholding in WGCNA. **b**-**c** WGCNA identifies modules significantly associated with METTL1 / WDR4

**Fig. 5:**
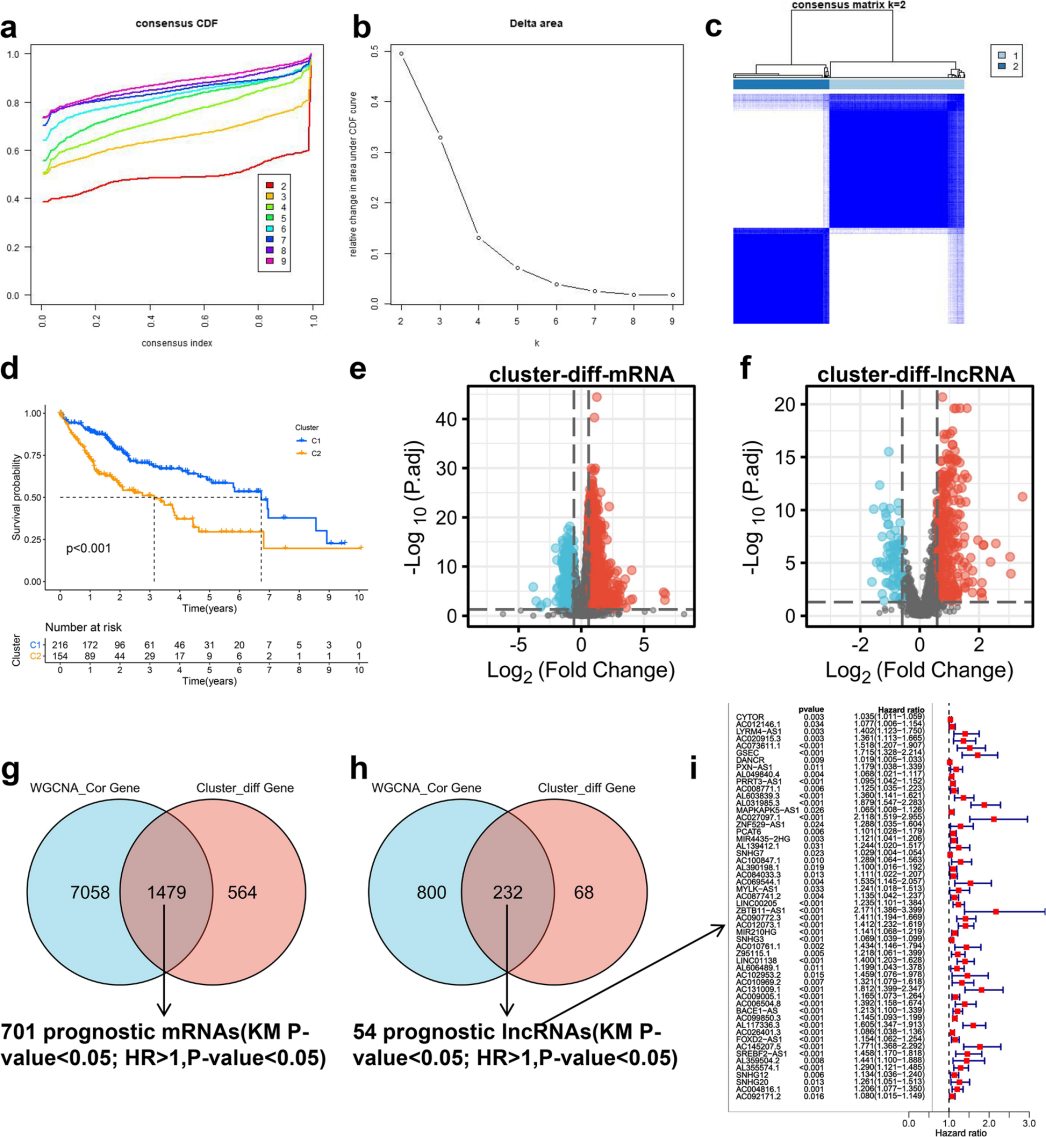
Acquisition of mRNAs and lncRNA closely related to HCC. **a** Cumulative distribution map of clustering consistency. **b** Clustering Delta Area map. **C** Clustering results of METTL1 / WDR4 on TCGA HCC samples. ( Overall survival curves showing differences in overall survival for C1 versus C2. **e**–**f** Volcano plots showing the differential mRNAs and lncRNAs between C1 and C2. **g**-**h** vnn plots of genes showing group differences co-intersection with genes in the WGCNA results. **i** Forest plot showing lncRNAs with poor prognosis for HCC patients

In this study, WGCNA was employed to construct a co-expression network of mRNA / lncRNA related to METTL1 / WDR4. The optimal soft thresholds for generating the co-expression networks of METTL1 / WDR4 associated mRNA / lncRNA were all 12 (Fig. 4a). Weighted cluster analysis was performed according to the optimal soft threshold. Finally, WGCNA of mRNA and lncRNA each generated five modules with significant positive correlations with METTL1 / WDR4 (*P* < 0.05) (Fig. 4b-c). Through the inclusion criteria (*r* > 0.4), RNAs in 2 MRAN modules (blue and yellow) and two lncRNA modules (brown and Turquoise) were selected for subsequent research in this study.This study constructed an unsupervised consensus clustering of TCGA HCC samples based on the METTL1 / WDR4 expression pattern for HCC classification. Based on CDF (Fig. 5a) and delta area (Fig. 5b), it could be well classified into two clusters (C1 and C2) when k = 2 (Fig. 5c). Overall survival curve results indicated that the overall survival time of C2 was significantly lower than that of C1 (*P* < 0.001) (Fig. 5d). Based on C1 and C2 subclusters, differential genes between C1 and C2 (|logfc|> 0.585, *P* < 0.05) were screened in this study. A total of 2043 upregulated mRNAs (Fig. 5e) and 300 lncRNA (Fig. 5f) were identified in this study. In addition, the mRNAs / lncRNAs with differences were significantly different from clinicopathological features (T, grade, and stage) between C1 and C2 (Additional file 1: Figure S4a-b). Subsequently, the present study intersected the above WGCNA results with the upregulated RNAs of the METTL1 / WDR4 subpopulation, and 1479 mRNAs (Fig. 5g) and 232 lncRNAs (Fig. 5h) were obtained. Based on the univariate Cox regression analysis, 701 mRNAs out of 1479 mRNAs showed poor prognosis for HCC patients (Additional file 3). While 54 lncRNAs out of 232 lncRNAs exhibited poor prognosis for HCC patients (Fig. 5i). This study identified 701 mRNAs and 54 lncRNAs as closely related mRNAs and lncRNA in HCC. Furthermore, these RNAs' GO and KEGG results correlated with tumors' development (Additional file 1: Figure S4c).

### Construction of a METTL1 / WDR4 associated mRNA and lncRNA risk signature

**Fig. 6:**
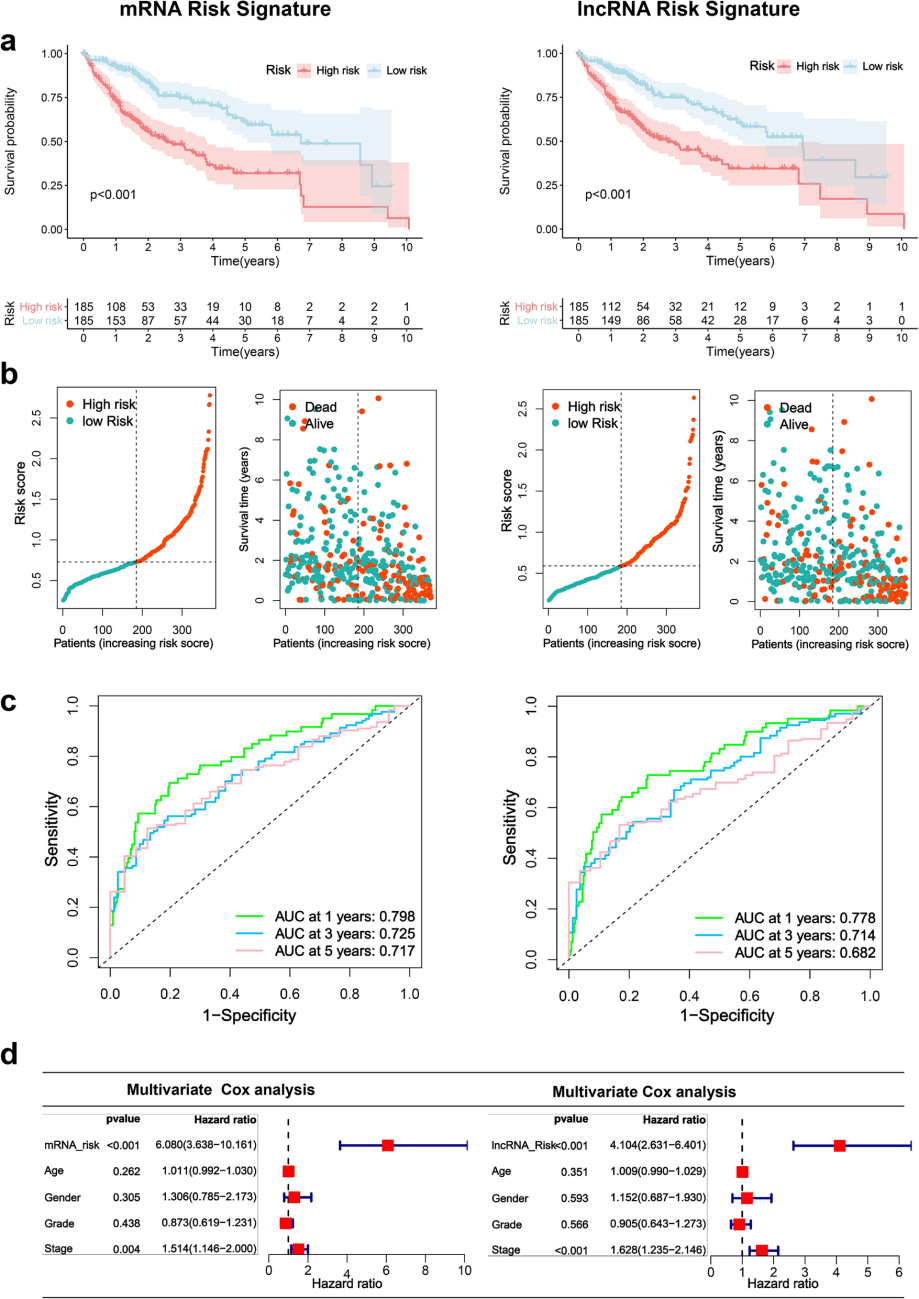
Construction of a METTL1 / WDR4 associated mRNA and lncRNA risk signature. **a** Overall survival curves showing survival differences between high and low risk groups of the training set. **b** High and low risk score median values and survival status distribution. **c** Time-dependent ROC curve. **d** Multivariate Cox analysis

This study employed lasso regression analysis to identify the most suitable prognostic mRNAs/ lncRNA for HCC patients based on HCC closely related mRNAs/ lncRNA (Additional file 1: Figure S5 a-b). A total of 19 mRNAs and 10 lncRNAs were determined in this study for constructing the optimal risk signature. In addition, these 19 mRNAs and 10 lncRNAs were upregulated and significantly positively correlated with METTL1 / WDR4 in HCC (Additional file 1: Figure S6a-b). The risk score was calculated as follows: Risk score(mRNA) = (0.00006* SMOX exp.) + (0.00218* ANXA2 exp.) + (0.00814 * FAM217B exp.) + (0.02334 * YARS exp.) + (0.05994 * TRNP1 exp.) + (0.00003 * GNAZ exp.) + (0.02987* EFNA4 exp.) + (0.00061 * NDRG1 exp.) + (0.12008 * KIAA1841 exp.) + (0.0299 * UCK2 exp.) + (0.00011 * KPNA2 exp.) + (0.00153* CAD exp.) + (0.009 * CDCA8 exp.) + (0.0026 * G6PD exp.) + (0.00716 * KIF20A exp.) + (0.00392 * PSRC1 exp.) + (0.02786 * MEX3A exp.) + (0.00548 * BCORL1 exp.) + (0.00107 * YBX1 exp.); Risk score(lncRNA) = (0.00897* ZNF529-AS1 exp.) + (0.02945* PRRT3-AS1 exp.) + (0.02796 * AL031985.3 exp.) + (0.01593 * MYLK-AS1 exp.) + (0.010709 * DANCR exp.) + (0.00608 * MIR210HG exp.) + (0.06631 * LINC01138 exp.) + (0.14516 * AC131009.1 exp.) + (0.05086 * AC099850.3 exp.) + (0.10909 * ZBTB11-AS1 exp). The HCC samples were divided into high-risk and low-risk groups according to the median value of the risk score. Based on the mRNA and lncRNA risk signature, the high-risk group had a significantly worse overall survival than the low-risk group (*P* < 0.05) (Fig. 6A), and there were more deaths in the high-risk group (Fig. 6b). The area under the curve (AUC) at 1, 3, and 5 years in the time-dependent ROC curve for the mRNA risk signature was 0.77, 0.718, and 0.702, respectively (Fig. 6c). The AUCs at 1, 3, and 5 years in the time-dependent ROC curves of the lncRNA risk signature were 0.778, 0.714, and 0.682, respectively (Fig. 6c). Principal component analysis (PCA) and t-SNE results indicated that HCC patients with different risks were able to be well classified into two clusters (Additional file 1: Figure S7). Moreover, multivariate Cox analysis indicated that the mRNA and lncRNA risk signatures were independent poor prognostic factors for HCC (Fig. 6d).

### Construction of a Nomogram prediction model

**Fig. 7:**
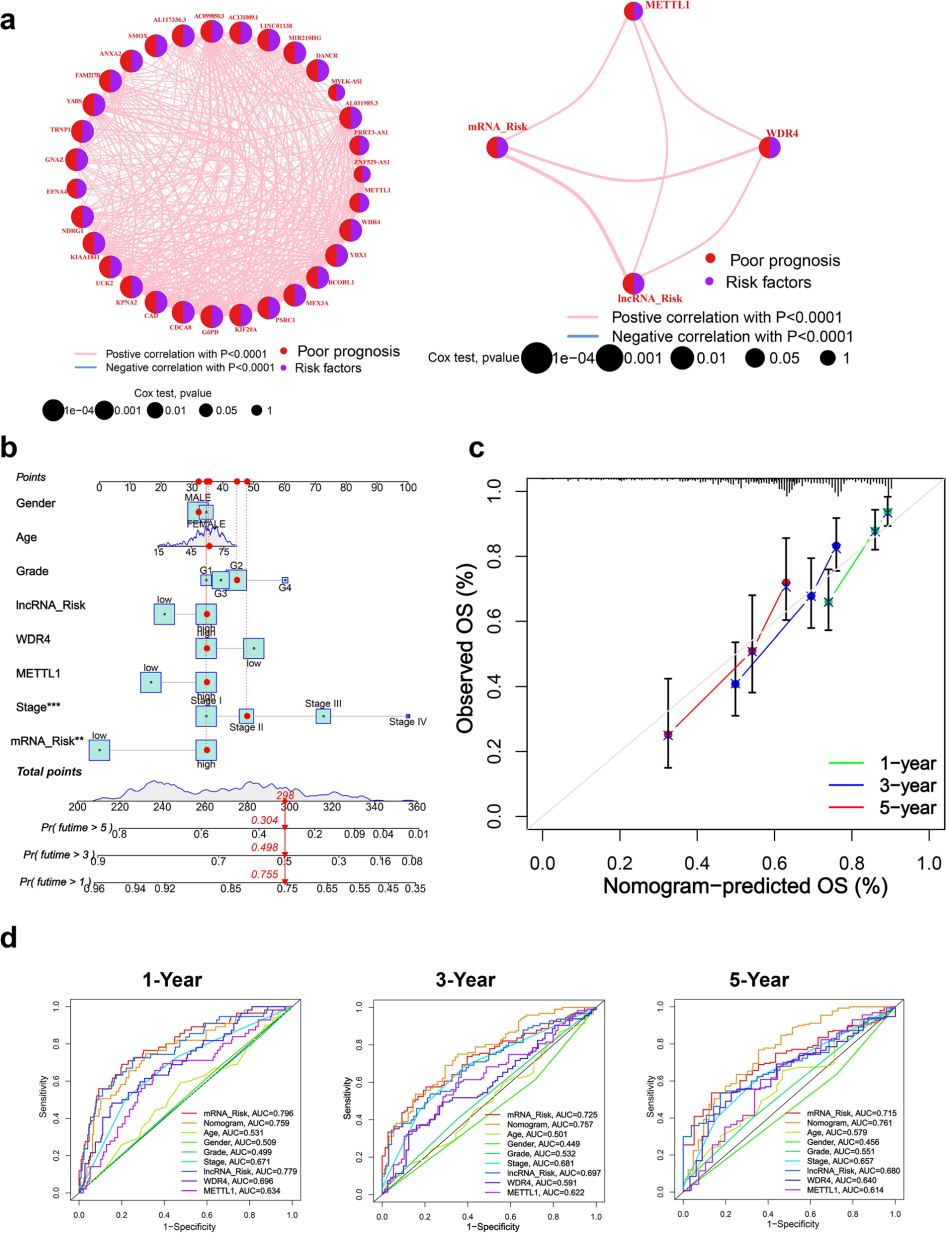
Construction of a Nomogram prediction model. **a** Risk network plot of METTL1 / WDR4 with mRNA / lncRNA risk signature. **b** Nomogram prediction model. **c** Calibration curve. (d) ROC curves show AUC values for individual parameters

We first constructed a risk network diagram of METTL1 / WDR4 with mRNA / lncRNA risk signature genes in this study. In this study, we found that METTL1 / WDR4 were significantly positively correlated with mRNA / lncRNA risk signature genes, and all of them were poor prognostic factors for HCC (Fig. 7a). Subsequently, in this study, the mRNA / lncRNA risk signature, METTL1 / WDR4 expression, age, gender, grade, and stage were included in the multivariate regression analysis. A Nomogram prediction model was constructed to further evaluate the OS prediction of HCC patients (Fig. 7b). The calibration curves indicated that the Nomogram prediction model was able to predict the 1, 3, and 5-year OS of HCC patients with better accuracy (Fig. 7c). The ROC results indicated that the mRNA risk signature (1-year AUC = 0.796) was optimal in predicting 1-year OS; However, the nomogram prediction model (3-year AUC = 0.757 and 5-year AUC = 0.761) was optimal in predicting OS at 3 and 5 years (Fig. 7d).

### Differences between METTL1 / WDR4 and mRNA / lncRNA risk signatures and clinicopathological features

**Fig. 8:**
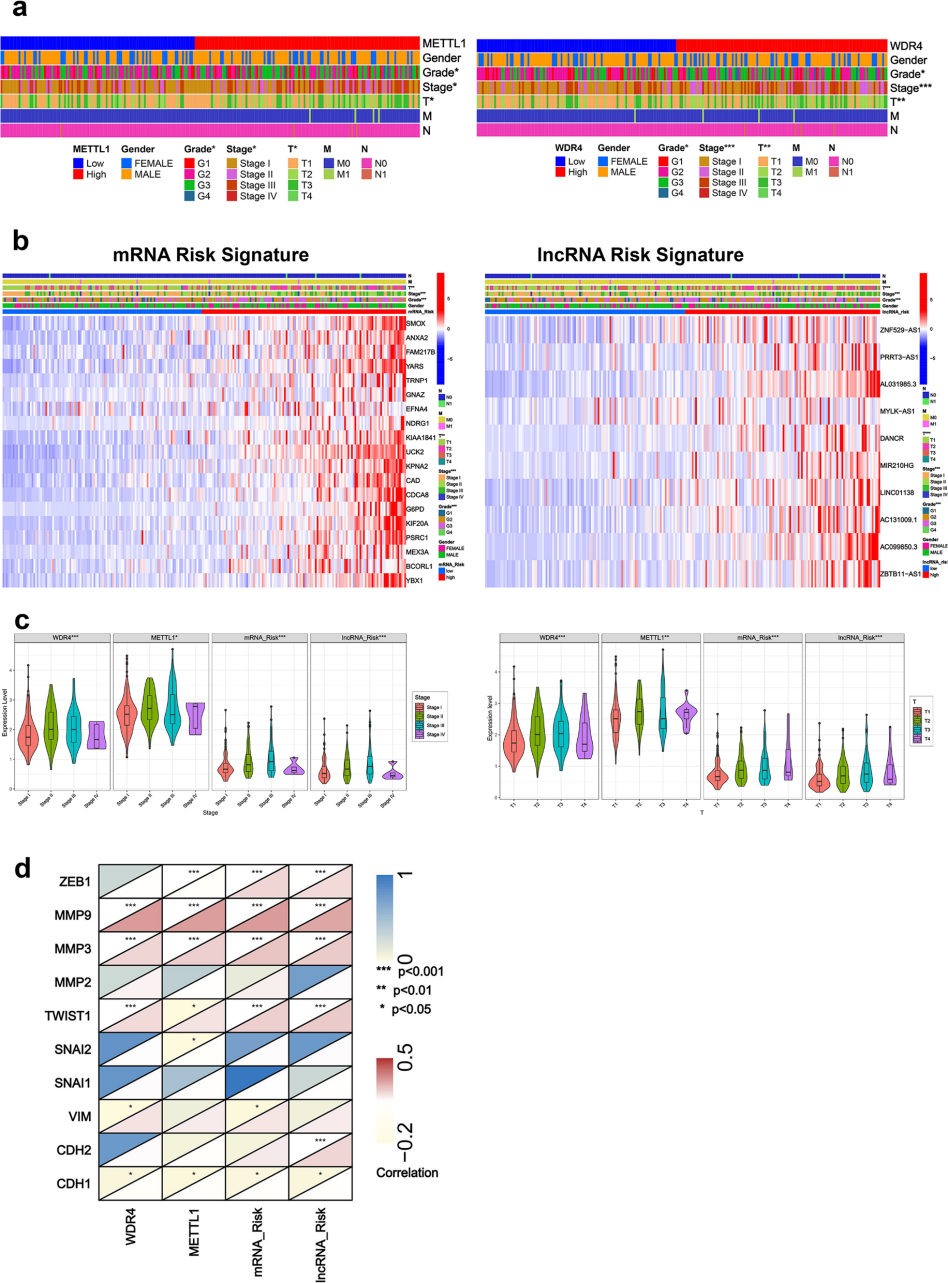
Differences between METTL1 / WDR4 and mRNA / lncRNA risk signatures and clinicopathological features. **a**-**b** Associations between high and low METTL1 / WDR4 and mRNA / lncRNA risk signatures and multiple clinicopathological features. **c** The expression of METTL1 / WDR4 and the mRNA / lncRNA risk scores varied among stages and invasion depth (T). **d** Correlation of METTL1 / WDR4 and mRNA / lncRNA risk signatures with EMT core genes

This study revealed the differences between the METTL1 / WDR4 and mRNA / lncRNA risk signatures and clinicopathological features. Grade, stage, and invasion depth (T) were significantly different between high and low METTL1 / WDR4 expression groups and high and low mRNA / lncRNA risk groups (*P* < 0.05) (Fig. 8a-b). In addition, the changes in METTL1 / WDR4 expression and mRNA / lncRNA risk scores were significantly different at different stages and invasion depth (T) (*P* < 0.05). (Fig. 8c). Since the progression of the HCC stage and invasion depth (T) has a close relationship with epithelial-mesenchymal transition (EMT). In this study, we found that the METTL1 / WDR4 and mRNA / lncRNA risk signatures were significantly associated with multiple EMT core genes, including EMT Suppressors (CDH1) and EMT promoters (MMP9, MMP3, and TWIST1) (Fig. 8d) (*P* < 0.05).

### Differences of HCC major gene mutations between high and low METTL1 / WDR4 expression groups and high and low mRNA / lncRNA risk groups

**Fig. 9:**
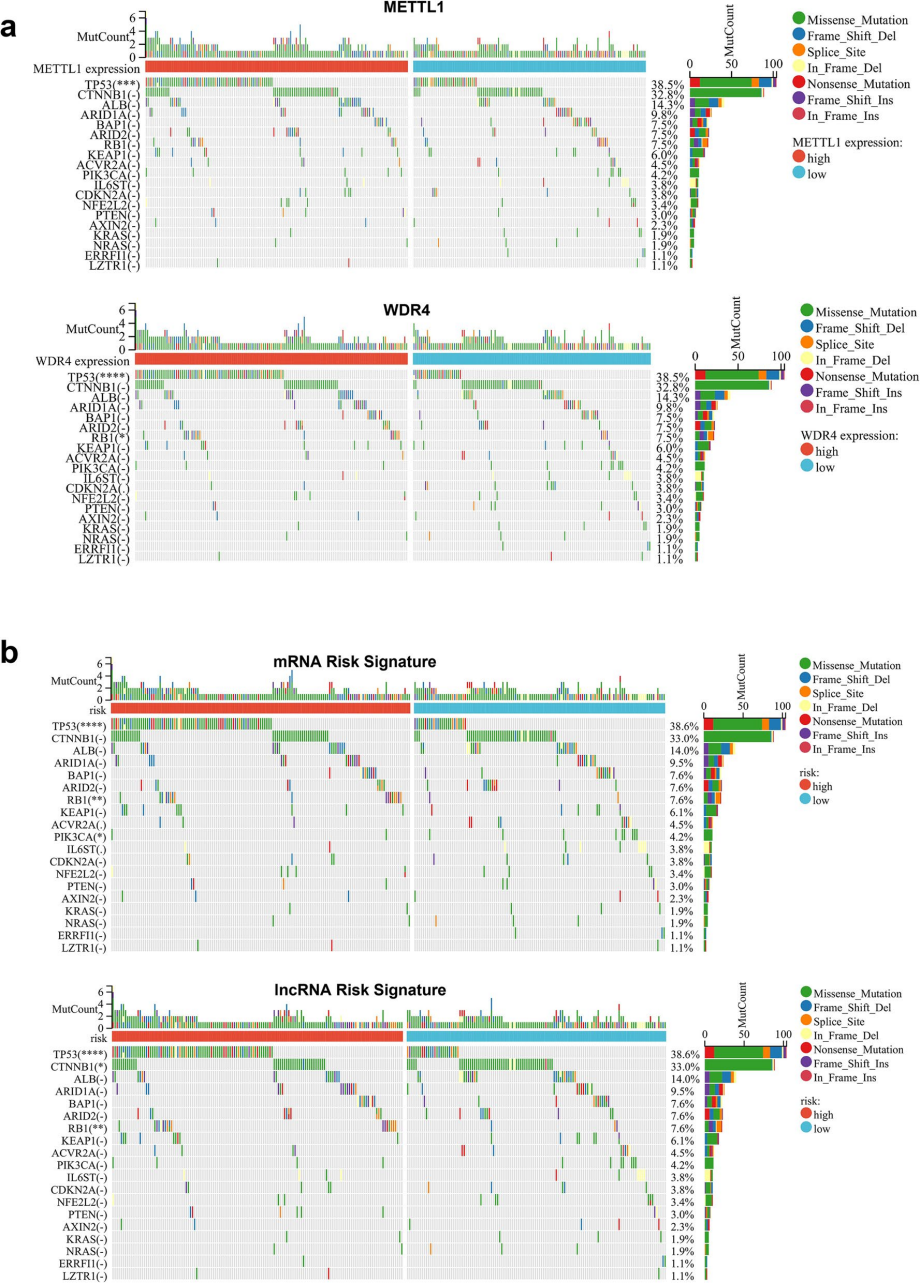
Differences of HCC major gene mutations between high and low METTL1 / WDR4 expression groups and high and low mRNA / lncRNA risk groups. **a**-**b** Waterfall plots showed the mutation differences of the major mutated genes in 20 HCCs between high and low METTL1 / WDR4 expression groups and high and low mRNA / lncRNA risk groups

This study compares the mutational differences between high and low in 20 genes with significant mutations in HCC. Among the METTL1/WDR4 high and low expression groups, TP53 was significantly (*P* < 0.05) mutated in the METTL1/WDR4 high expression group, and RB1 was significantly (*P* < 0.05) mutated in the WDR4 high expression group (Fig. 9a). In the mRNA/lncRNA risk signature, TP53 and RB1 were significantly more mutated in the high-risk group, while PIK3CA was significantly less mutated in the high-risk group (*P* < 0.05) (Fig. 9b).

### METTL1/WDR4 and mRNA risk signature genes were expressed to varying degrees in various cells

**Fig. 10:**
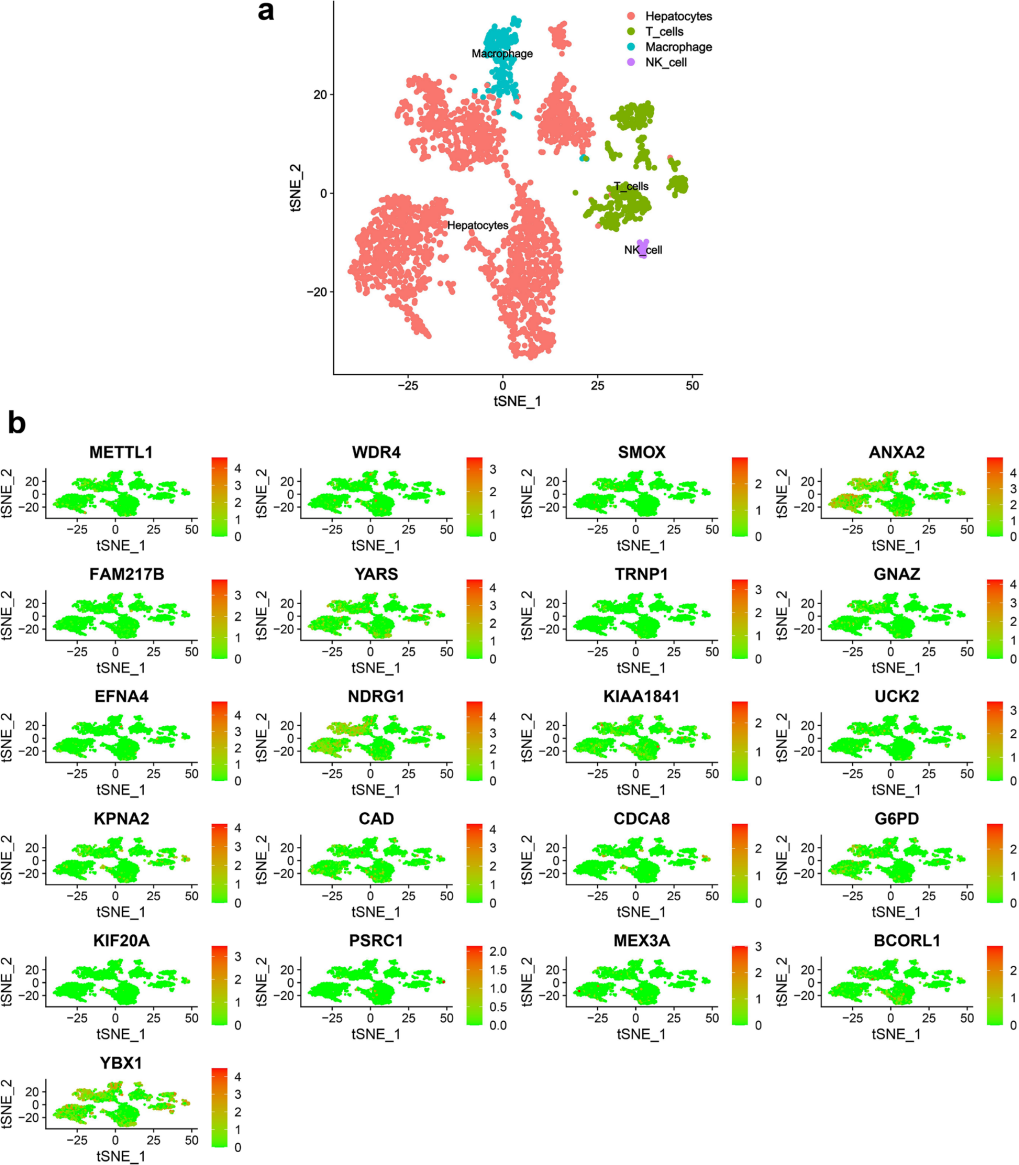
METTL1/WDR4 and mRNA risk signature genes were expressed to varying degrees in various cells. **a** T-SNE plot of 3199 cell subpopulations from 4 HCC samples. **b** Distribution of METTL1 / WDR4 and 19 mRNA risk signature genes in four cell types (liver parenchymal cells, macrophages, NK cells, and T cells)

In this study, after quality control and data filtering on the HCC single-cell sequencing dataset GSE146115 (*n* = 4), we obtained gene expression profiles for 3199 high-quality cells (Additional file 1: Figure S8a). In this study, the detection depth was not correlated with mitochondrial genes but was proportional to the number of qualifying genes tested (r = 0.8) (Additional file 1: Figure S8b). In addition, 1500 variable genes were used for subsequent cell fractionation and cell annotation (Additional file 1: Figure S8c). After PCA dimensionality reduction (Additional file 1: Figure S8d) and t-SNE treatment, 12 cell populations were identified in this study (Additional file 1: Figure S8e). Subsequently, this study annotated the cell populations. We obtained four cell populations (liver parenchymal cells, macrophages, NK cells, and T cells) (Fig. 10a). In this study, we found that METTL1 / WDR4 and 19 mRNA risk signature genes were expressed in these four types of cells to different degrees (Fig. 10b).

### Construction of an immune escape-associated PPI network composed of METTL1 / WDR4 and mRNA risk signature genes

**Fig. 11:**
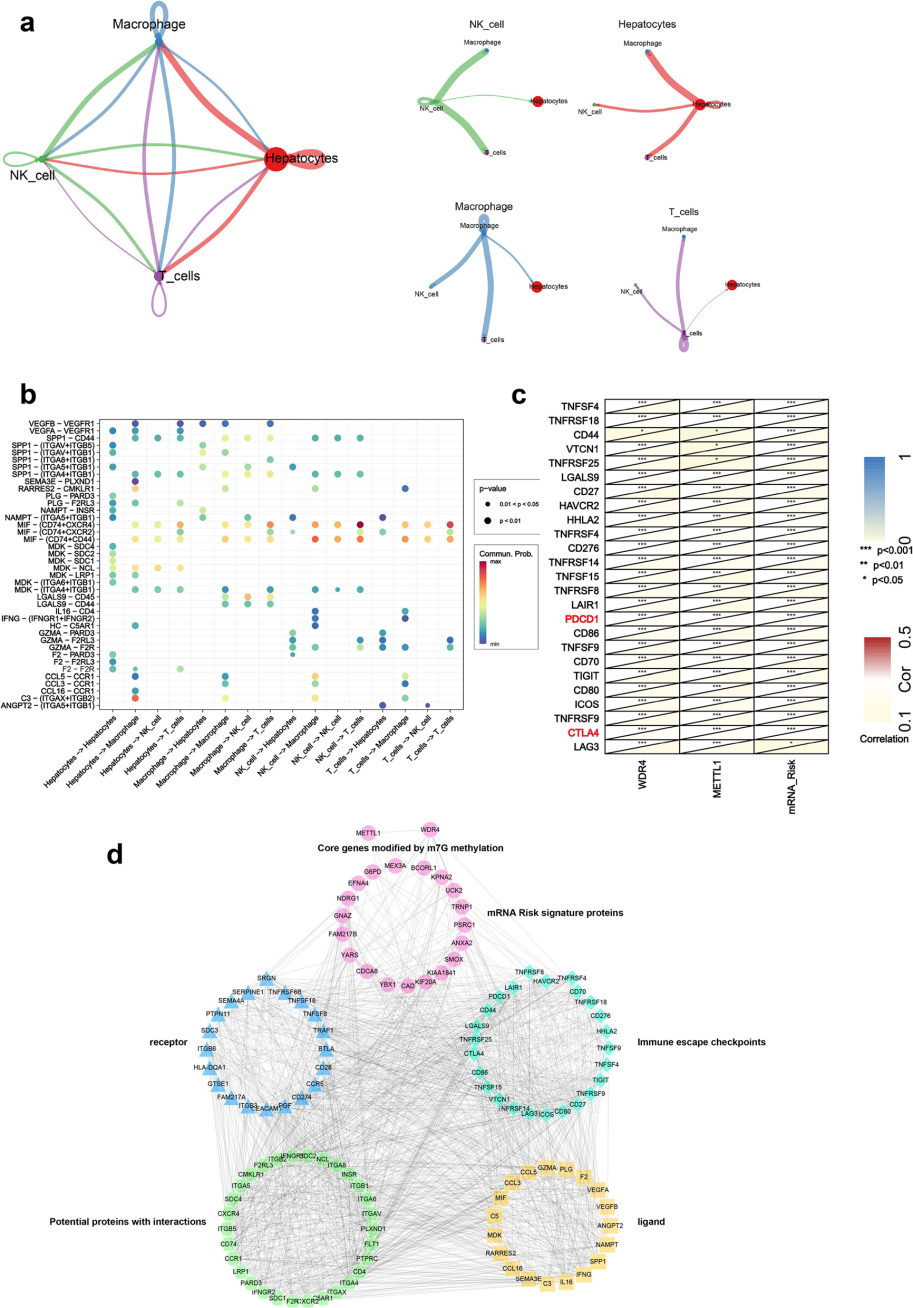
Construction of an immune escape-associated PPI network composed of METTL1 / WDR4 and mRNA risk signature genes. **a** Cellular communication between liver parenchymal cells, macrophages, NK cells, and T cells. **b** Receptors and ligands between liver parenchymal cells, macrophages, NK cells, and T cells. **c** Correlation analysis between METTL1 / WDR4 and mRNA risk signature with 26 immune escape-related checkpoints. **d** The immune escape-related PPI network, including METTL1 / WDR4 (Circles), 19 mRNA risk signature proteins (Circles), four cellulars (liver parenchymal cells, T cells, NK cells, and macrophages) ligands (rectangles), and receptors (Hexagon), 26 immune escape related proteins (Diamonds), and potential proteins with interactions with them (Triangles)

The present study found crosstalk among liver parenchymal cells, macrophages, NK cells, and T cells through communication between cells (Fig. 11a). Subsequently, we predicted the receptors and ligands between the four types of cells (Fig. 11b). Since liver parenchymal cells, macrophages, NK cells, and T cells are closely related to immune escape. In addition, we found a significant positive correlation between the METTL1 / WDR4 / mRNA risk signature and 26 immune escape-related checkpoints, including the classical immune escape checkpoints PDCD1 and CTLA4 (Fig. 11c). Therefore, in this study, an immune escape-related PPI network was constructed, including METTL1 / WDR4 (Circle), 19 mRNA risk signature proteins (Circle), four cellulars (liver parenchymal cells, T cells, NK cells, and macrophages) ligands (rectangle) and receptors (Hexagon), 26 immune escape related proteins (Diamond) and potential proteins with interactions with them (Triangle) (Fig. 11d).

## Discussion

HCC accounts for 80% – 90% of all liver cancers [1]. Many factors cause HCC, including genetics, HBV / HCV, alcohol consumption, radiation [17]. Unfortunately, HCC patients have low cure rates and high mortality rates, highlighting the urgent need for practical prognostic markers and therapeutic targets for this disease.

Aberrant expression of m7G methylation regulators has been linked to tumor progression. One of the best-characterized regulators of m7G methylation is METTL1 and WDR4, which can form a METTL1 / WDR4 complex to regulate the m7G methylation modification of multiple RNAs [18]. The METTL1 / WDR4 complex currently promotes tumor development by mediating tRNA m7G methylation modification. For example, Hui Han et al. showed that the METTL1 / WDR4 complex promotes esophageal squamous cell carcinoma by activating the RPTOR/ULK1 autophagy pathway through tRNA m7G methylation modification [19]. Similarly, Xiaoling Ying et al. showed that the METTL1 / WDR4 complex is required for bladder cancer progression by regulating the EGFR/EFEMP1 axis via tRNA m7G methylation modification [20]. Jieyi Ma et al. also found that METTL1 / WDR4 plays a role in promoting lung cancer progression through tRNA m7G methylation modification [10]. Additionally, the METTL1/WDR4 complex is involved in the m7G methylation modification of miRNAs, promoting their production [21]. In a study by Luca Pandolfini et al., downregulation of METTL1 expression in lung cancer cells resulted in reduced let-7 miRNA expression and decreased cell migration [21].

In this study, we investigated the role of METTL1 and WDR4 in HCC and explored the effects of METTL1 / WDR4 and related RNAs (mRNA and lncRNA) on the prognosis and immune escape of HCC patients. In this study, we demonstrated that down-regulation of METTL1 / WDR4 reduced the proliferation, migration, and invasion of HCC cells in vitro. Furthermore, we observed that METTL1 and WDR4 expression were upregulated in HCC tissues, and their expression was positively correlated. High expression of METTL1 and WDR4 was associated with decreased survival time in OS, DSS, and PFI survival curves, and METTL1/WDR4 were identified as independent poor prognostic factors in HCC. Previous studies have shown that knockdown of METTL1 reduced tRNA m7G methylation modification [22]. In another study, MYC promoted CCNB1 translation and, in turn, proliferation and metastasis of HCC cells by targeting WDR4 [22]. Recent studies have shown that blocking the METTL1-TGF-β2-PMN-MDSC axis can reduce the recurrence of HCC, further highlighting the importance of METTL1/WDR4 in HCC progression. [23]. Therefore, METTL1 / WDR4 are closely associated with HCC progression. Collectively, METTL1 / WDR4 promotes HCC initiation and progression, leading to poor prognosis in HCC patients.

Currently, mRNAs interacting with METTL1 or WDR4 have been rarely studied in HCC. METTL1 / WDR4 have been shown to modify mRNA with m7G methylation. METTL1 functions as an m7G methyltransferase to modify mRNA with m7G methylation, and WDR4 is required to facilitate the binding of the METTL1 / WDR4 complex to target mRNAs [24]. Studies have shown that METTL1 promotes the production of vascular endothelial growth factor A (VEGFA) through m7G methylation modification, which increases angiogenesis [25]. In this study, we constructed the optimal METTL1 / WDR4 associated mRNA and lncRNA risk signature based on multiple algorithms, including 19 mRNAs and 10 lncRNAs. We found that 11 mRNAs in the mRNA risk signature acted as oncogenes,, including SMOX [26], ANXA2 [27], GNAZ [28], EFNA4 [29], NDRG1 [30], UCK2 [31], KPNA2 [32], CAD [33], CDCA8 [34], KIF20A [35], YBX1 [36]. The remaining seven mRNAs have not been experimentally studied to date, including FAM217B, YARS1, TRNP1, KIAA1841, G6PD, PSRC1, MEX3A, BCORL1. Long non-coding RNAs RNAs (lncRNA) are RNA molecules composed of more than 200 nucleotides and cannot encode proteins [37]. It has been demonstrated that lncRNA is closely associated with the occurrence of HCC [38]. Among numerous RNA modifications, lncRNAs can be extensively modified by N6 methylation (m6A) regulators [39]. However, the link with m7G methylation modification, especially with METTL1 / WDR4 acting lncRNA, has not been studied. In the METTL1 / WDR4 associated lncRNA risk signature, MYLK-AS1 promotes HCC progression by regulating the miR-424-5p/E2F7 axis and VEGFR-2 signaling pathway [40]; LINC01138 can interact with PRMT5 and promote metastasis and proliferation of HCC by enhancing its protein stability [41]. There are no pilot studies for the other seven lncRNA risk signature genes, including ZNF529-AS1, PRRT3-AS1, AL031985.3, DANCR, MIR210HG, AC131009.1, and ZBTB11-AS1. In addition, this study's mRNA and lncRNA risk scores could discriminate between patients at different risks. mRNA and lncRNA risk scores were independent poor prognostic factors for HCC patients. Therefore, the METTL1 / WDR4 associated mRNA and lncRNA risk signatures constructed in this study have essential effects on HCC progression.

As METTL1 / WDR4 and mRNA / lncRNA risk signatures are independent poor prognostic factors for HCC, respectively. Therefore, 1 -, 3 -, and 5-year Nomogram prediction models were constructed in this study. Calibration and multiparametric ROC curves demonstrated this nomogram prediction model's high accuracy and validity. Studies have shown that the HCC stage and invasion depth (T) are poor prognostic factors for HCC patients [42]. In the present study, T and stage significantly differed between the high and low groups. The changes in METTL1 / WDR4 expression and mRNA / lncRNA risk score significantly differed in different stages and T. Moreover, EMT is not only one of the culprits promoting HCC progression but also an essential factor contributing to the poor prognosis of HCC [43]. In this study, we found that the METTL1 / WDR4 and mRNA / lncRNA risk signatures were significantly inversely correlated with the EMT suppressor (CDH1) but not with the EMT promoting factors (MMP9, MMP3, and TWIST1). Yang Yang et al. showed that by inhibiting the transcription of CDH1 and then promoting the progression of HCC [44]. In addition, upregulated MMP9, MMP3, and TWIST1 can promote metastasis of HCC and predict poor prognosis of HCC [45]. Therefore, in this study, the METTL1 / WDR4 and mRNA / lncRNA risk signatures had significant prognostic significance for HCC patients. In the mutation differential analysis of this study, TP53 mutations were increased in samples from high expression or high-risk groups of METTL1 / WDR4 and mRNA / lncRNA risk signature. One of the most striking TP53 mutations [46]. TP53 functions as a tumor suppressor in HCC under normal circumstances. However, once TP53 is mutated, mutant TP53 has a role in promoting HCC development [47]. Moreover, mutation of TP53 is one of the factors associated with poor prognosis in HCC patients [48]. RB1 mutations were increased in samples of high expression or high-risk groups for WDR4 and mRNA / lncRNA risk signature. RB1 negatively regulates cell cycle progression and functions as a tumor suppressor gene in HCC [49]. However, if RB1 is mutated will increase HCC incidence [50]. Therefore, TP53 and RB1 mutations are HCC risk factors. The above further illustrated the prognostic value of METTL1 / WDR4 and mRNA / lncRNA risk signature for HCC.

The tumor microenvironment (TME) plays a critical role in HCC progression and prognosis [51]. In this study, we utilized single-cell sequencing data to identify four distinct cell populations, including liver parenchymal cells, T cells, NK cells, and macrophages, that exhibit crosstalk. Further analysis revealed varying degrees of expression of METTL1/WDR4 and 19 mRNA risk signature genes in these cell types. Previous studies have demonstrated that receptors could increase the capacity of HCC immune escape by inhibiting T cells [52]. NK cells are the first line of defense against tumorigenesis and an essential component of innate immunity in humans [53]. The imbalance of NK cells by ligands may increase the risk of immune escape in HCC [54]. Macrophages are the most infiltrated immune cells of the tumor microenvironment, and tumor-associated macrophages can increase HCC immunosuppression and vascularization [55]. Our study found that METTL1 / WDR4 and mRNA risk signatures were significantly associated with 26 immune escape checkpoints, including the classical immune escape checkpoints T lymphocyte antigen 4 (CTLA4) and programmed cell death protein 1 (PDCD1). PDCD1 and CTLA4 can cause exhaustion of T cells and inhibit the release of T cell immune material [56]. Immune checkpoints can prevent immune over-activation, but in the tumor microenvironment, elevated immune checkpoint expression leads to the tumor microenvironment being in an immunosuppressive state [57–59]. Therefore, we constructed immune escape-related PPIs, including METTL1 / WDR4, 19 mRNA risk signature proteins, four cell receptor ligands (liver parenchymal cells, T cells, NK cells, and macrophages), 26 immune escape-related proteins and potential proteins with interactions with them. Our findings suggest that METTL1/WDR4 and 19 mRNA risk signature genes are associated with immune escape, and this PPI provides a basis for subsequent HCC immune escape studies.

Several limitations exist in this study. While we validated the expression of WDR4/METTL1 RNA levels using in vitro experiments and multiple datasets, their protein levels still require validation through in vitro experiments. Additionally, while we demonstrated the effect of WDR4/METTL1 on HCC using two kinds of cell function experiments, the mechanism underlying their actions remains unclear. Further experiments are therefore necessary to clarify the mechanism of WDR4/METTL1 on HCC. Moreover, the mRNA/lncRNA risk signature we constructed was based solely on TCGA-HCC data, and large-scale clinical samples are necessary to validate the stability and reliability of these two risk features.

In summary, the core genes (METTL1 and WDR4) and mRNA / lncRNA risk signature of the m7G methylation modification were independent poor prognostic factors for HCC. The nomogram prediction model constructed in this study was able to better predict the overall survival of HCC patients at 1, 3, and 5 years. The METTL1 / WDR4 and mRNA / lncRNA risk signatures were associated with clinicopathological features and major HCC gene mutations. Furthermore, the HCC immune escape-related PPIs we constructed provide a theoretical basis for subsequent immune escape mechanisms. Overall, our findings provide a foundation for identifying prognostic markers, therapeutic targets, and immune escape mechanisms in HCC.

## Conclusions

We analyzed METTTL1 and WDR4 and constructed their associated mRNA and lncRNA risk signatures, which may serve as survival predictors and potential predictive biomarkers for HCC. In addition, we constructed an immune escape-related PPI network that provides a basis for studying the mechanism of immune escape and searching for therapeutic targets.

## Supplementary Information

**Additional file 1.**

**Additional file 2.**

**Additional file 3.**

## Data Availability

The datasets generated and analysed during the current study are available in the TCGA repository (https://portal.gdc.cancer.gov), Genotype-Tissue Expression (GTEx) repository (https://www.gtexportal.org/home/index.html), International Cancer Genome Consortium(ICGC) repository (https://dcc.icgc.org/) and GEO database (https://www.ncbi.nlm.nih.gov/geo/).

## Abbreviations

METTL1: Methyltransferase 1
WDR4: WD repeat domain 4
TCGA: The Cancer Genome Atlas
GEO: GENE EXPRESSION OMNIBUS
GTEx: GenotypeTissue Expressiongtex
ICGC: International Cancer Genome Consortium
DSS: Disease-specific survival
PFS: Progression Free Surviva
EMT: Epithelial–mesenchymal transition
PPI: Protein–protein interaction
WGCNA: Weighted coexpression network analysis
Lasso: Least absolute shrinkage and selection operator
m7G: N7 methylguanosine
RPTOR: Regulatory associated protein of MTOR complex 1
ULK1: Unc-51 like autophagy activating kinase 1
VEGFA: Vascular endothelial growth factor A
SMOX: Spermine oxidase
ANXA2: Annexin A2
GNAZ: G protein subunit alpha z
EFNA4: Ephrin A4
NDRG1: N-myc downstream regulated 1
UCK2: Uridine-cytidine kinase 2
KPNA2: Karyopherin subunit alpha 2
CAD: Carbamoyl-phosphate synthetase 2, aspartate transcarbamylase, and dihydroorotase
CDCA8: Cell division cycle associated 8
KIF20A: Kinesin family member 20A
YBX1: Y-box binding protein 1
FAM217B: Family with sequence similarity 217 member B
YARS1: Tyrosyl-tRNA synthetase 1
TRNP1: TMF1 regulated nuclear protein 1
G6PD: Glucose-6-phosphate dehydrogenase
PSRC1: Proline and serine rich coiled-coil 1
MEX3A: Mex-3 RNA binding family member A
BCORL1: BCL6 corepressor like 1
