# Supplementary material for: M7G methylated core genes (METTL1 and WDR4) and associated RNA risk signatures are associated with prognosis and immune escape in HCC
Source: BMC Med Genomics. 2023 Aug 1;16:179. doi: 10.1186/s12920-023-01614-8 (PMC10394781; doi:10.1186/s12920-023-01614-8)
Supplement: Supplementary file 1 — Additional file 1. [file 12920_2023_1614_MOESM1_ESM.docx]

**
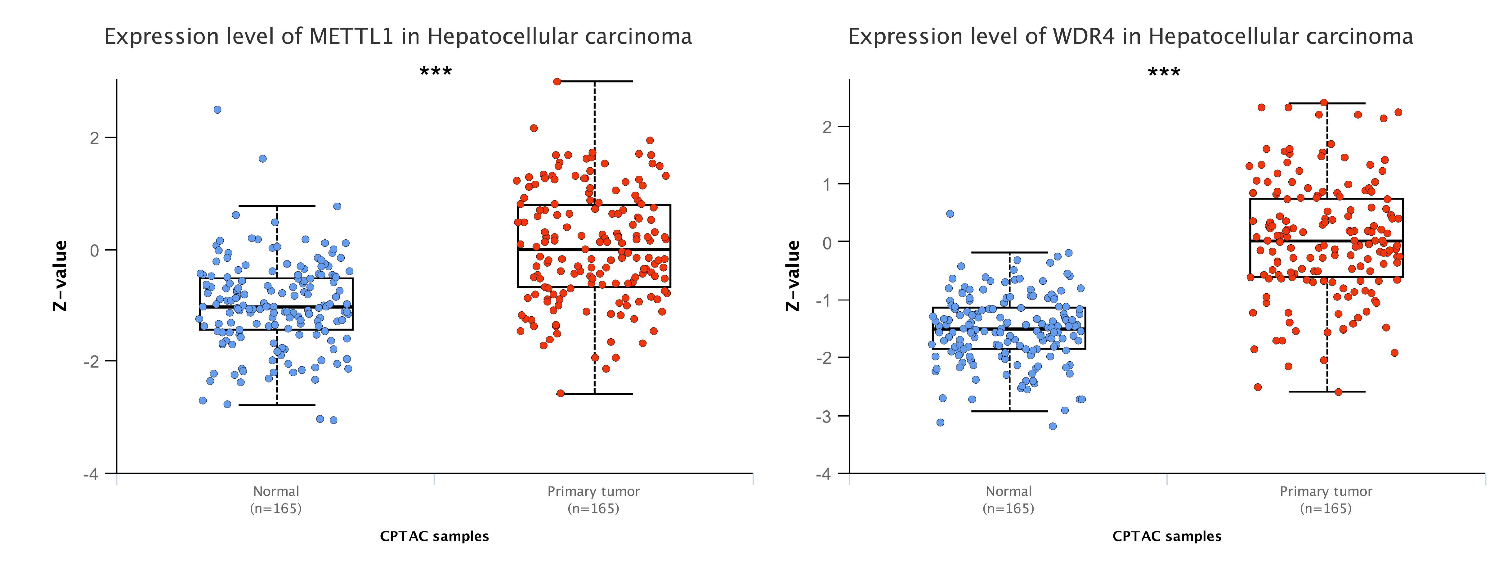
**

**Supplementary Figure 1** Based on the UALCAN database, the expression of METTL1 and WDR4 at the protein level was assessed.

**
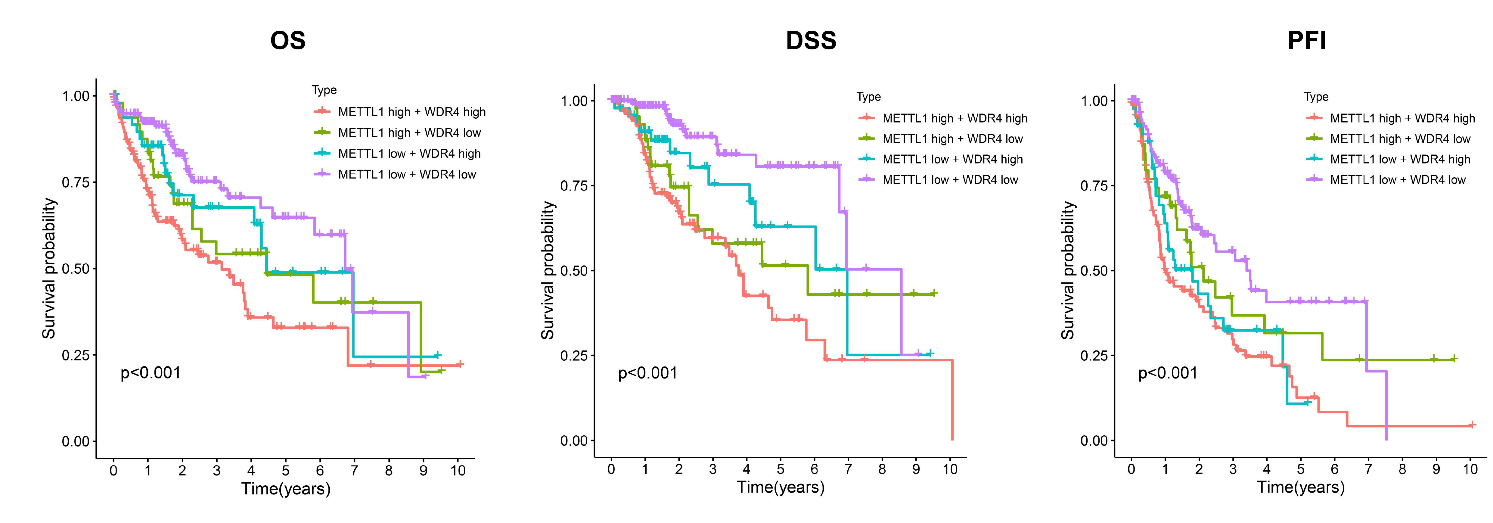
**

**Supplementary Figure 2** Overall survival (OS), disease-specific survival (DSS), and progression-free interval (PFI) curves for METTL1 + WDR4.

**
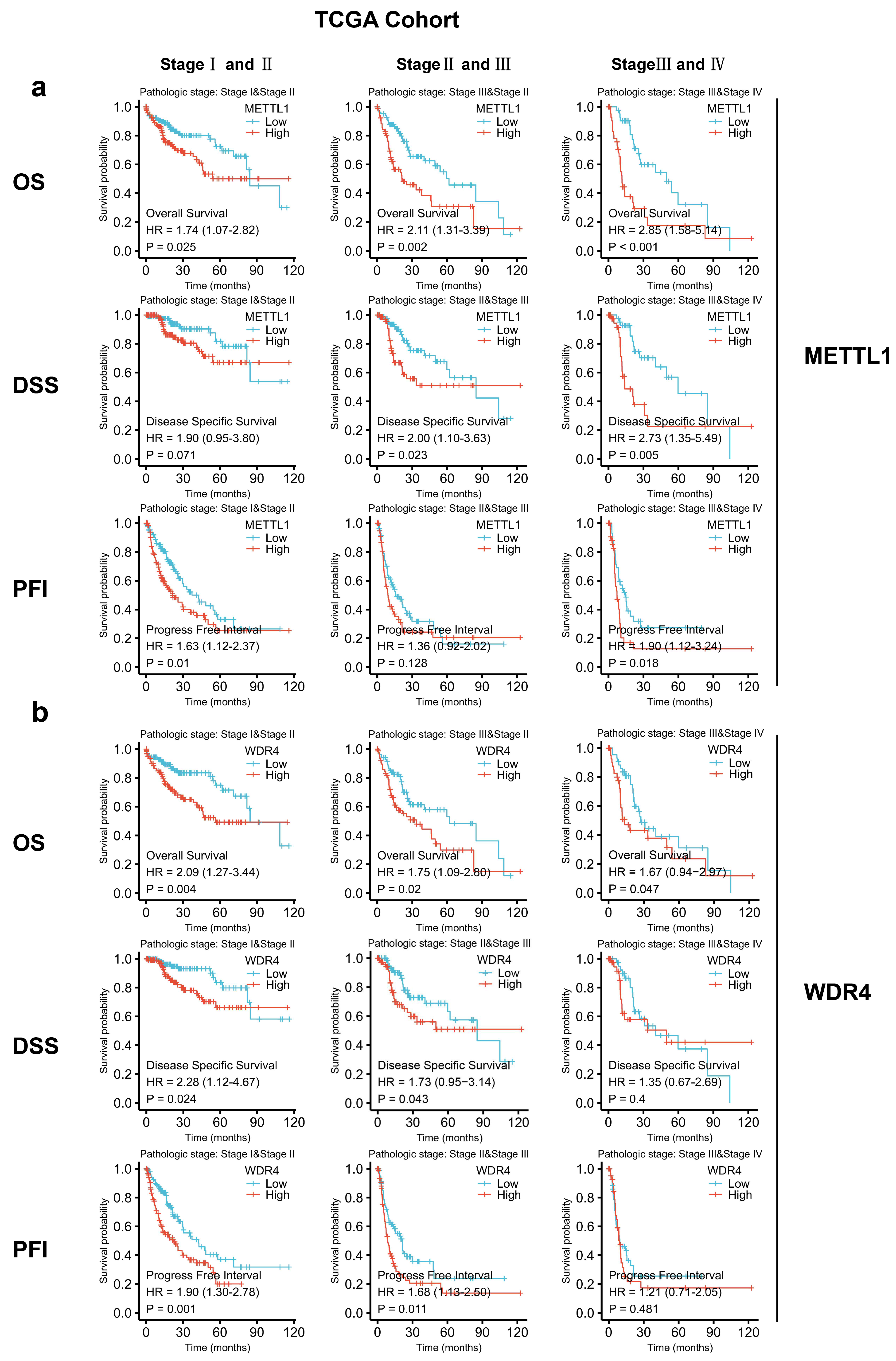
**

**Supplementary Figure 3** Overall survival curves by HCC stage. (a-b) Survival curves of METTL1 and WDR4 in stage subtypes.


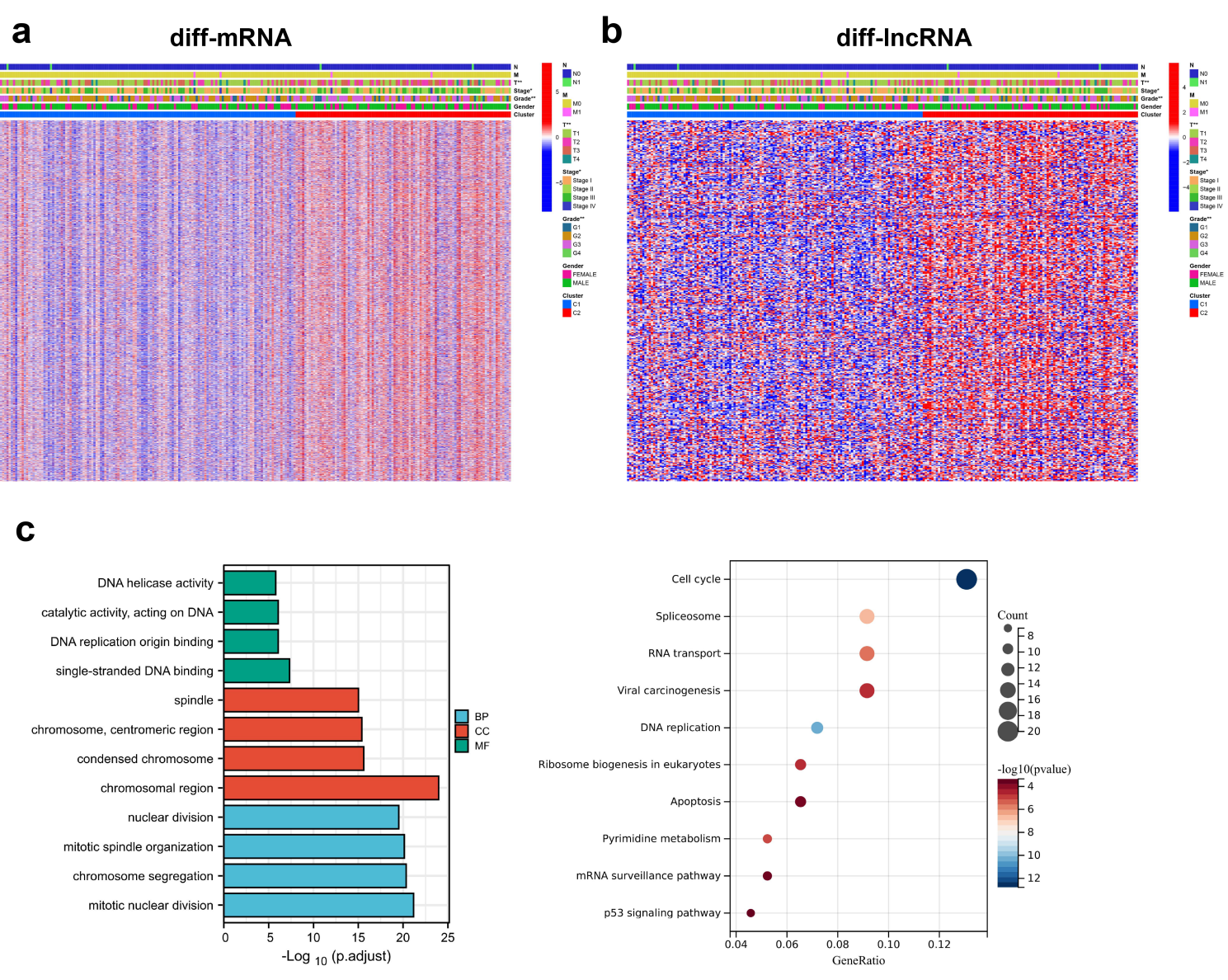


**Supplementary Figure 4** Clinicopathological differences and GO / KEGG analysis of differential genes between C1 and C2. (a-b) The mRNAs / lncRNAs with differences were significantly different from clinicopathological features (T, grade, and stage) between C1 and C2. (c) GO and KEGG.


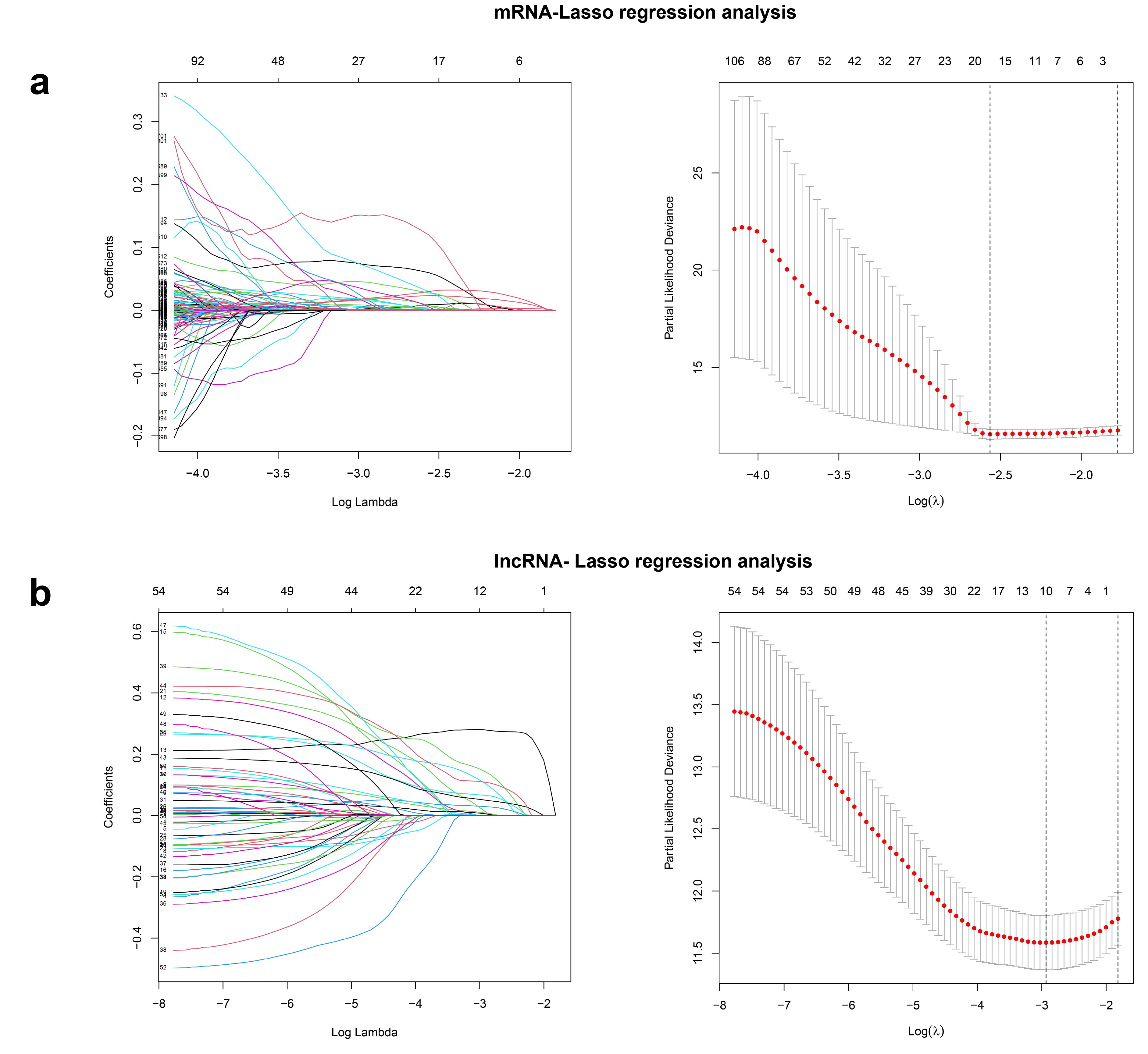


**Supplementary Figure 5** (a-b) Lasso regression analysis.


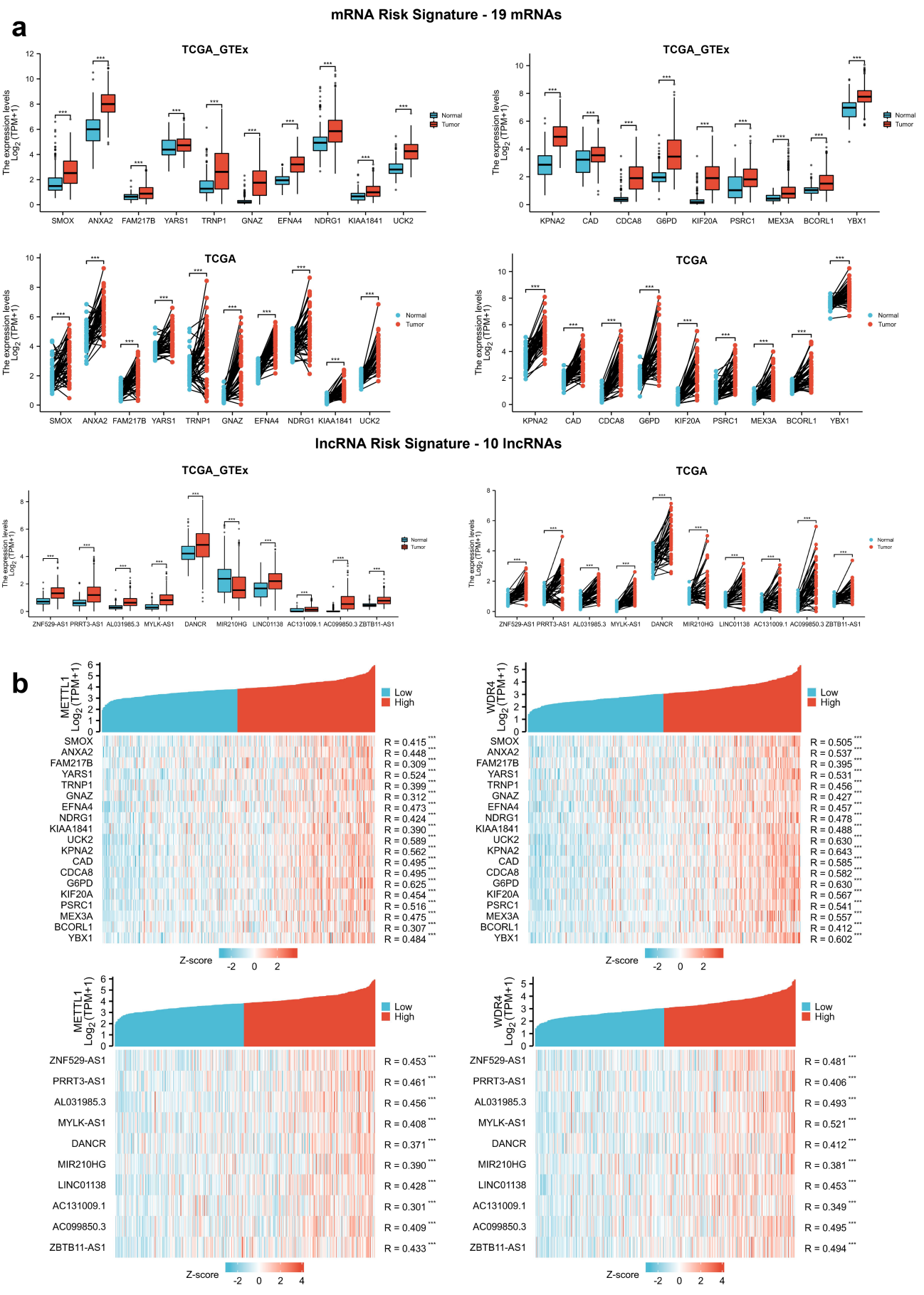


**Supplementary Figure 6** Differential analysis and co-expression analysis of mRNA / lncRNA risk signature genes. (a) Differential analysis of 19 mRNAs and 10 lncRNAs. (b) Heatmap of co expression of 19 mRNAs and 10 lncRNAs with METTL1 / WDR4.


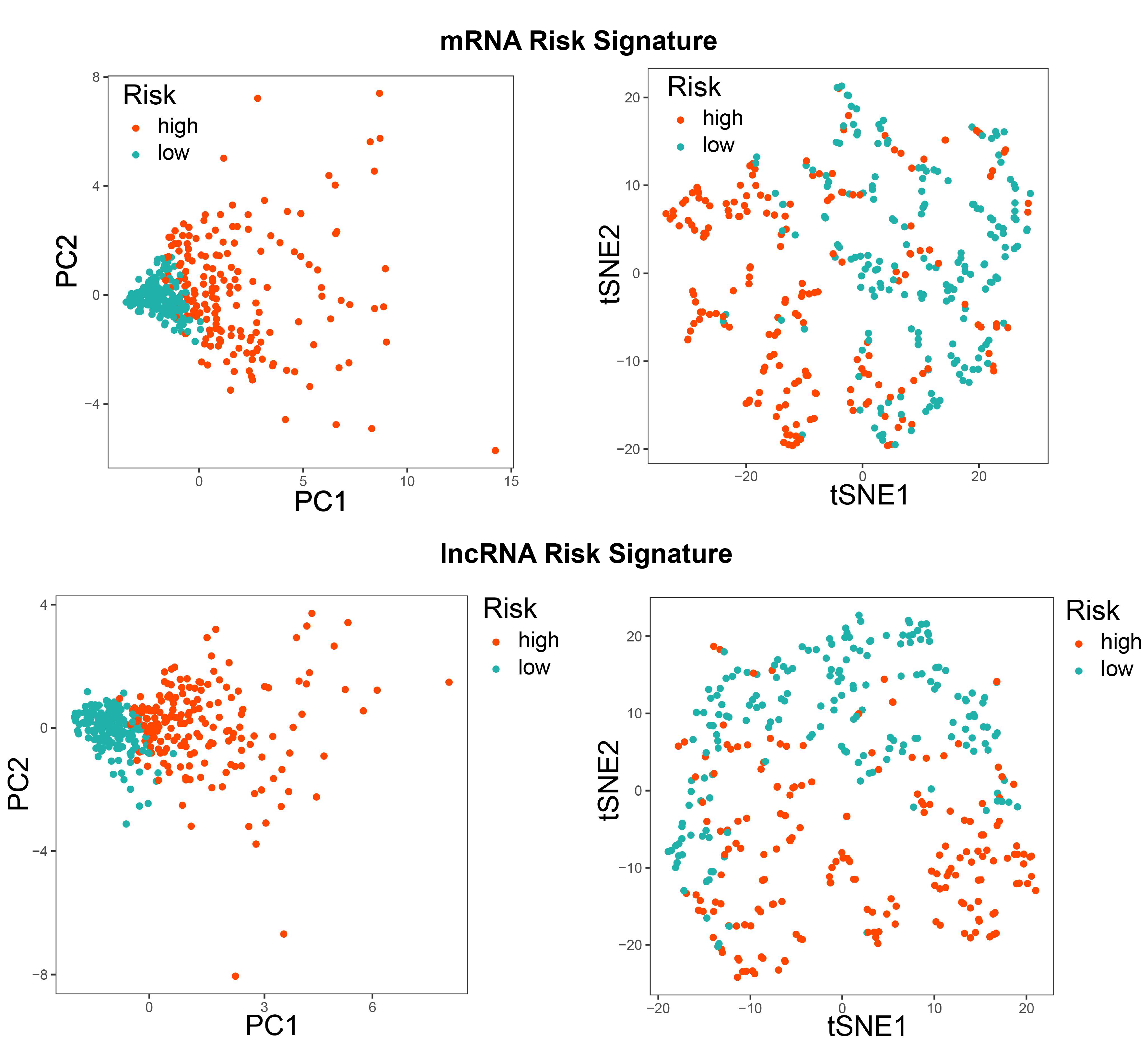


**Supplementary Figure 7** Principal component analysis (PCA) and t-SNE.


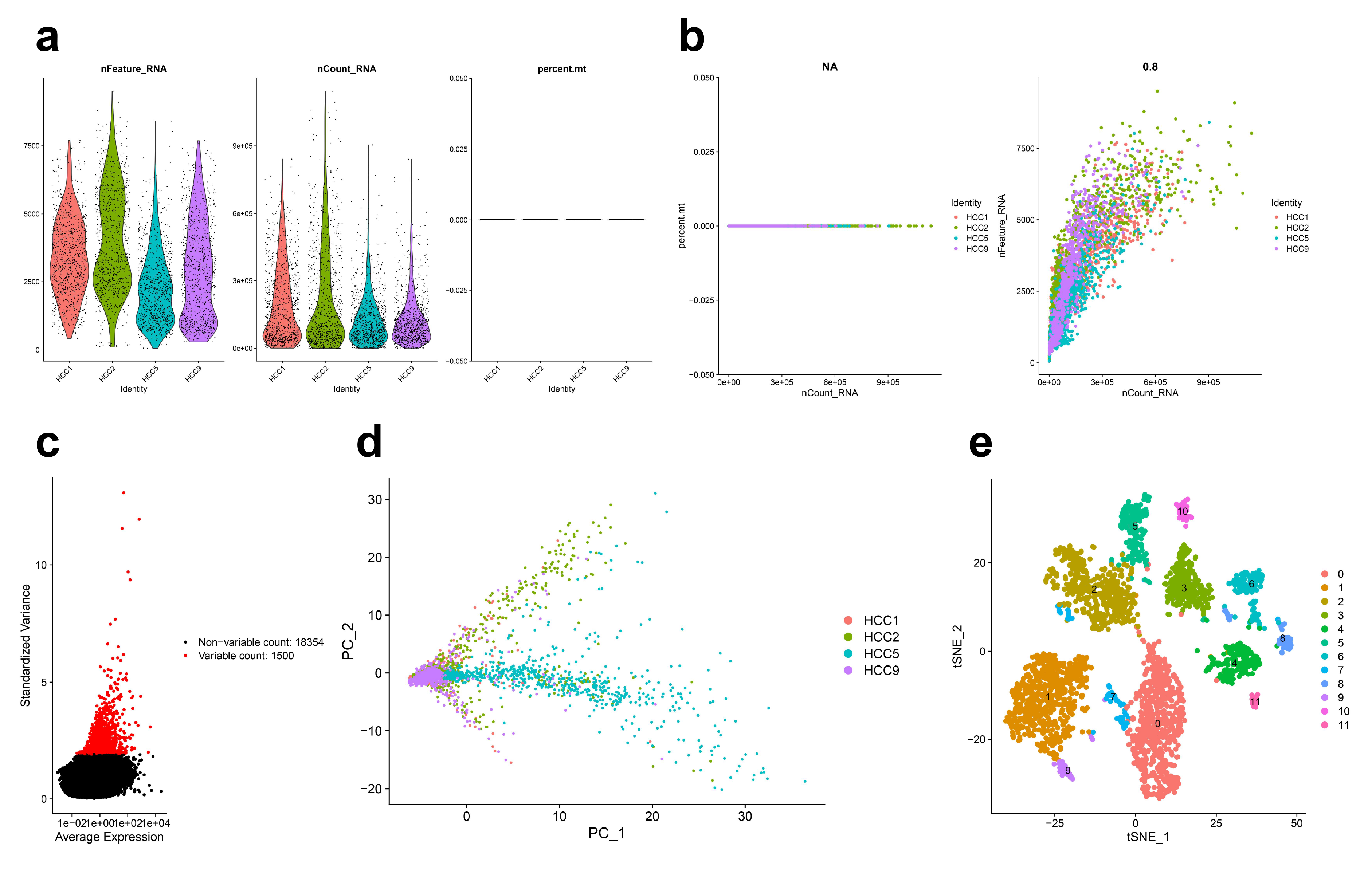


**Supplementary Figure 8** Quality control and filtering of single cell sequencing results. (a) Gene expression of 3199 high quality cells from four HCC samples. (b) The detection depth was not correlated with mitochondrial genes but was proportional to the number of qualifying genes tested. (c) The top 1500 variable genes with large, standardized variances were selected for subsequent analysis. (d) Principal component analysis (PCA). (e) After t-SNE treatment, 12 cell populations were identified.
