# Supplementary material for: M7G methylated core genes (METTL1 and WDR4) and associated RNA risk signatures are associated with prognosis and immune escape in HCC
Source: BMC Med Genomics. 2023 Aug 1;16:179. doi: 10.1186/s12920-023-01614-8 (PMC10394781; doi:10.1186/s12920-023-01614-8)
Supplement: Supplementary file 2 — Additional file 2. [file 12920_2023_1614_MOESM2_ESM.docx]

| Study | Platform | Tumour Group(n) | Normal Group(n) | Total(n) | PMID |
| --- | --- | --- | --- | --- | --- |
| GSE112790 | GPL570 | 183 | 15 | 198 | 30598371 |
| GSE121248 | GPL570 | 70 | 37 | 107 | 17975138 |
| GSE14520 | GPL3921 | 228 | 217 | 445 | 32502310 |
| GSE25097 | GPL10687 | 268 | 249 | 517 | 21949730 |
| GSE29721 | GPL570 | 10 | 10 | 20 | 21747116 |
| GSE41804 | GPL570 | 20 | 20 | 40 | 23426277 |
| GSE45436 | GPL570 | 93 | 41 | 134 | 24160375 |
| GSE54236 | GPL6480 | 81 | 80 | 161 | 36276500 |
| GSE57957 | GPL10558 | 211 | 80 | 291 | 25093504 |
| GSE60502 | GPL96 | 18 | 17 | 35 | 25376302 |
| GSE62232 | GPL570 | 81 | 10 | 91 | 25822088 |
| GSE64041 | GPL6244 | 60 | 65 | 125 | 27499918 |
| GSE76427 | GPL10558 | 115 | 57 | 172 | 29117471 |
| GSE84402 | GPL570 | 14 | 14 | 28 | 28810927 |
| GSE115018 | GPL20115 | 12 | 12 | 24 | 30364632 |
| GSE12941 | GPL5175 | 10 | 10 | 20 | 20388846 |
| GSE136247 | GPL17586 | 39 | 30 | 69 | 33747361 |
| GSE65484 | GPL4091 | 50 | 14 | 64 | 26697315 |
| GSE77314 | GPL9052 | 50 | 50 | 100 | 27119355 |
| GSE45114 | GPL5918 | 24 | 25 | 49 | 24564407 |
| GSE17856 | GPL6480 | 50 | 44 | 94 | 20380719 |

**Table S1.** 21 GEO dataset details.
